# Post-vaccination serum cytokines levels correlate with breakthrough influenza infections

**DOI:** 10.1038/s41598-023-28295-8

**Published:** 2023-01-20

**Authors:** Weichun Tang, Hang Xie, Zhiping Ye, Angelia A. Eick-Cost, Mark Scheckelhoff, Courtney E. Gustin, Jay H. Bream, Ewan P. Plant

**Affiliations:** 1 Laboratory of Pediatric and Respiratory Viral Disease, Office of Vaccine Research and Review, CBER, FDA, Silver Spring, MD USA; 2 Armed Forces Health Surveillance Division, Defense Health Agency, Silver Spring, MD USA; 3 Department of Molecular Microbiology and Immunology, Johns Hopkins Bloomberg School of Public Health, Baltimore, MD USA; 4 Graduate Program in Immunology, Johns Hopkins School of Medicine, Baltimore, MD USA

**Keywords:** Vaccines, Immunology, Cytokines, Infectious diseases, Inflammation, Vaccines

## Abstract

Post-vaccination cytokine levels from 256 young adults who subsequently suffered breakthrough influenza infections were compared with matched controls. Modulation within the immune system is important for eliciting a protective response, and the optimal response differs according to vaccine formulation and delivery. For both inactivated influenza vaccine (IIV) and live attenuated influenza vaccines (LAIV) lower levels of IL-8 were observed in post-vaccination sera. Post-vaccination antibody levels were higher and IFN-γ levels were lower in IIV sera compared to LAIV sera. Subjects who suffered breakthrough infections after IIV vaccination had higher levels of sCD25 compared to the control group. There were differences in LAIV post-vaccination interleukin levels for subjects who subsequently suffered breakthrough infections, but these differences were masked in subjects who received concomitant vaccines. Wide variances, sex-based differences and confounders such as concomitant vaccines thwart the establishment of specific cytokine responses as a correlate of protection, but our results provide real world evidence that the status of the immune system following vaccination is important for successful vaccination and subsequent protection against disease.

## Introduction

Vaccination is intended to induce an immune response that leads to subsequent protection from disease. For many vaccines, including inactivated influenza vaccine (IIV), there is a correlation between antibody titers elicited by the vaccine and vaccine efficacy. Knowledge about the role played by other parts of the immune system is limited and more information is needed to provide a framework for understanding the biological mechanisms driving vaccine efficacy^1,2^. For example, live attenuated influenza vaccines (LAIVs) have been licensed based on efficacy without a correlate of protection (CoP). In seasons when vaccine efficacy is unusually low, it is more challenging to identify possible causes without correlates. When a CoP is available, as in the case of the IIVs, it is easier to identify other immune signals that track with the correlate, but these are often quantified with respect to antibody titers not vaccine efficacy. In addition, differences in innate immune status prior to vaccination also affect antibody responses to vaccination^3,4^. Here we investigate post-vaccination cytokine levels in subjects that subsequently suffered influenza infections and compare these to matched control groups.

Influenza viruses cause respiratory disease that results in significant seasonal morbidity and mortality in humans^5^. Influenza viruses affect other mammalian and avian species and occasionally jump from one host species to another but, for the most part, the genera currently afflicting humans are influenza A (subtypes H1N1 and H3N2) and influenza B. Vaccines are available that provide protection against these subtypes. However, the viruses continually evolve, and changes in the antigenically dominant viral hemagglutination (HA) protein led to a decline in protection. Currently available vaccines require yearly updates so that the antigenic components better match the circulating strains.

IIV and LAIV are manufactured differently and have different routes of administration. LAIV are administered intranasally where the live attenuated virus engages the immune system in mucosal tissues. Although there are indications of localized inflammation and increased antibody secreting cells after vaccination^6,7^, LAIV does not have a CoP. In contrast, most IIV are administered intramuscularly, and the antigenic components of the vaccine are processed in proximal lymph nodes. Antibody-secreting plasmablasts are prevalent the following week and elevated levels of activated B cells persist for several weeks^8^. These different methods of engaging the immune system both result in protection against disease through different (but perhaps not exclusive) mechanisms. The protection elicited by IIV correlates with antibodies that target the major viral glycoprotein, hemagglutinin. However, it has been shown that some cytokine levels are also associated with changes in antibody levels. For example, Wang et al.^9^ found that post-vaccination IL-10 levels correlated with antibody increase. Similarly, Krakauer and Russo^10^ observed lower IL-10 levels 30 days post-vaccination for IIV recipients who failed to reach protective antibody titers, but the difference didn’t reach significance in their study.

The immune mechanisms underlying LAIV vaccine effectiveness remain poorly understood. A marker of inflammation, CXCL10 (IP-10), has been shown to increase after LAIV vaccination^6^. Both the innate and humoral arms of the immune system respond to LAIV but engagement of each arm varies depending on the recipients age and prior exposure to influenza^11,12^. The innate response is guided by pattern recognition receptors that detect virus and the engagement of T-cells by the antigen presenting cells. This leads to the induction of cytokines and chemokines from T helper 1 (Th1) cells (TNF, IFN-γ, IL-2), Th2 cells (IL-4, IL-5, IL-13) and T follicular helper (T_FH_) cells (CXCL10)^13^. Although LAIV leads to an increase in influenza-specific interferon producing cells it has not been possible to correlate interferon, or other individual immune markers, with protection^14^. Influenza antibody titers are more pronounced toward strains encountered in the first decade of life^15^. The 2009 pdmH1N1 strain differed from the preceding seasonal H1N1 strains and was more closely related to contemporary swine viruses^16^. The presence of cross-protective antibodies in older adults, and a greater T-cell response after vaccination compared to younger adults demonstrate the importance of both arms of the immune system in an older population^17,18^.

Randomized clinical trials involving tens of thousands of subjects are necessary for demonstrating vaccine efficacy but have limited ability to link vaccine mechanism and effectiveness. Here we turn the traditional approach on its head by first identifying breakthrough infections in a population and then linking those to stored serum samples drawn post-vaccination. Cytokines in these samples were compared to matched controls. Soluble (s)CD25 serum levels correlated with breakthrough infections for subjects receiving IIV, and IL-6 levels correlated with breakthrough infections for subjects receiving LAIV. Subgroup analyses were performed to assess the impact of sex and concomitant vaccinations. For subjects that suffered breakthrough infections, regardless of vaccine modality and antibody titer, changes in post-vaccination serum cytokine levels were identified that correlated with subsequent infection, indicating that vaccine efficacy could be improved with better understanding of patient post-vaccination immune status.

## Results

### Differences in post-vaccination cytokine response for IIV and LAIV

**Figure 1:**
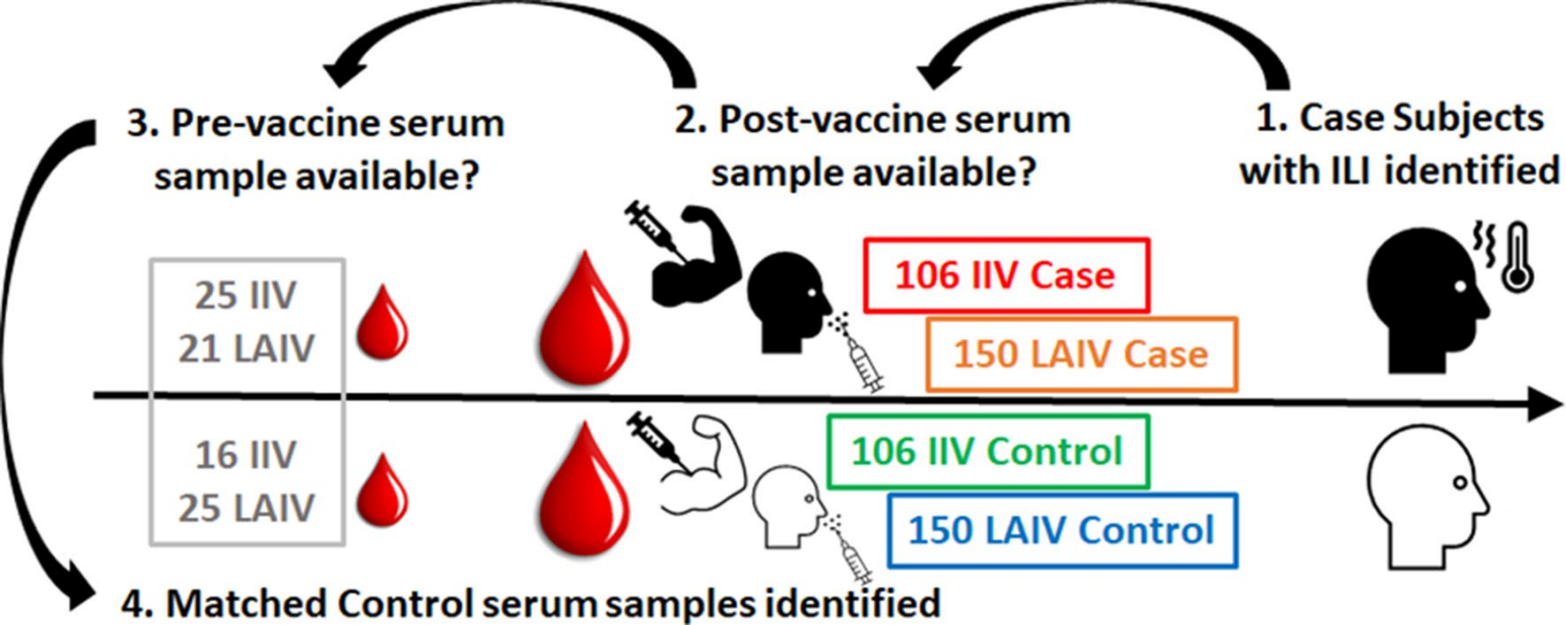
Overview of experimental design. First, case subjects with an influenza diagnosis during the 2009/10 influenza season were identified. Second, stored sera samples taken 1–21 days post-vaccination (IIV, inactivated influenza vaccine, or LAIV, live attenuated influenza vaccine) were selected. Third, where available, sera samples drawn within 6 months prior to vaccination were selected. Fourth, post-vaccination samples from matched control subjects with no record of ILL were selected, along with pre-vaccination samples where available. For most analyses the 87 pre-vaccination samples were treated as one group and compared to each of the four post-vaccination groups.

**Figure 2:**
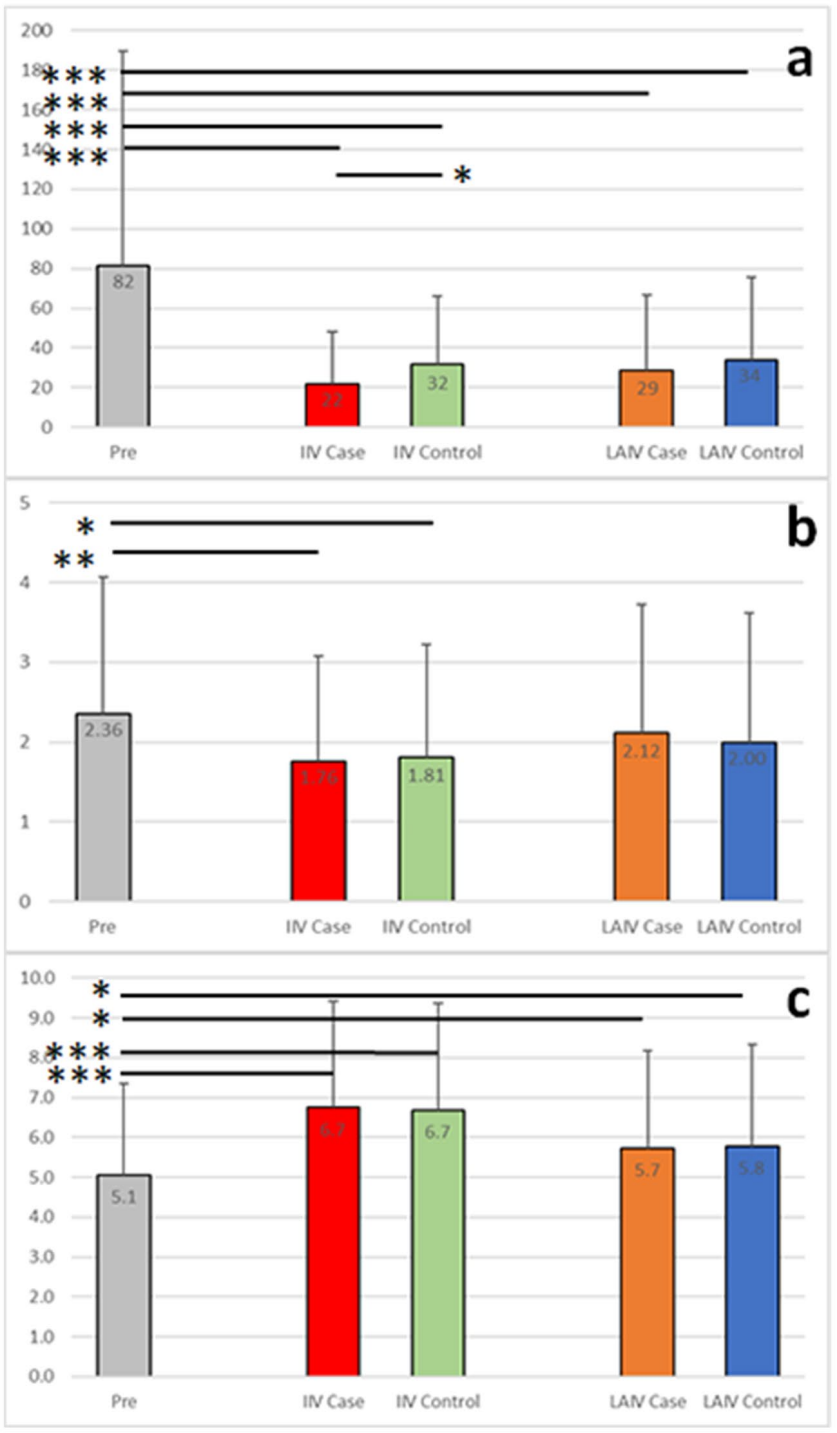
Comparison of pre-vaccination and post-vaccination cytokine and antibody levels. Serum cytokine measurements (pg/mL) are shown for IL-8 (**a**) and IFN-γ (**b**). Hemagglutination inhibition (HI) titers (log2) toward the A/California/07/2009 strain are shown in (**c**). The mean value for each group is shown with standard deviations. *p-value < 0.05; **p-value < 0.01; ***p-value < 0.001.

The Department of Defense (DoD) routinely collects and stores serum samples from service members and these are linked to medical surveillance and administrative health care data^19^. Serum samples are used in this prospective study to compare cytokines from vaccine recipients who subsequently suffered breakthrough infections with matched healthy subjects. There were 106 samples associated with IIV breakthrough infections and 150 samples associated with LAIV breakthrough infections (Supplementary Table 1). The same number of samples from matched individuals were identified and included in the study. From these 512 subjects 87 were identified with serum samples obtained within 6 months prior to vaccination (pre-vaccination samples). We compared the mean pre-vaccination cytokine levels from a subset of the subjects with the post-vaccination levels from four groups; IIV Case, IIV Control, LAIV Case and LAIV Control (Fig. 1 and Supplementary Table 1). We first sought to determine if changes in cytokine levels observed in our cohorts were comparable with existing studies. Although high levels of inter-subject variability are observed, the sample size in each group resulted in the detection of several significant differences. The mean levels for IL-8 and TNF-α are lower in all groups with either vaccine modality (Fig. 2, Supplementary Table 2 and Supplementary Fig. 1). The significance is more notable for IL-8 (p < 0.001 for all groups) than for TNF-α (p < 0.05 for the two IIV groups and the LAIV Case group but no significance for the LAIV Control group). We and others have reported reduced post-vaccination levels for IL-8 and TNF-α^20–22^. Martin et al.^23^ reported downregulation of IL-8 gene expression in PBMCs after vaccination with either IIV or LAIV, and an association between decreased serum TNF-α and lack of seroconversion after LAIV vaccination. The mean serum levels were also lower in all post-vaccination groups for IFN-γ, IL-6, IL-10 and BAFF (Fig. 2, Supplementary Table 2 and Supplementary Fig. 1) but only reached levels of significance for some groups (described with respect to breakthrough infections below). Serum IL-6, IL-1β, IFN-γ and TNF-α levels have been shown to drop below baseline levels in mice 24–48 h after receipt of inactivated influenza vaccines by McDonald et al.^24^. Pre-vaccination antibody titers were observed for both the seasonal H1N1 vaccine strain (A/Brisbane/59/2007) and the pandemic H1N1 strain (A/California/07/2009) (Fig. 2 and Supplementary Fig. 1). This is in line with prior vaccination or exposures and the observed spread of the 2009 pandemic H1N1 virus in the USA by late 2009^25^. Higher post-vaccination log2 HI titers for both A/Brisbane/59/2007 and A/California/07/2009 were observed the IIV Case and Control groups and to a lesser extent for the LAIV Case and Control groups (Fig. 2 and Supplementary Fig. 1). These results are in line with other studies and indicate the suitability of the prospective study design to investigate differences in post-vaccination cytokine levels.

We observed different trends in post-vaccination cytokine levels associated with each vaccine modality. For example, although mean IFN-γ levels trended lower for all four post-vaccination groups, the difference from the pre-vaccination mean was significant for the IIV groups but not the LAIV groups (Fig. 2). We observed that IL-1β and CXCL10 post-vaccination levels trended higher for both LAIV groups but not the IIV groups (Supplementary Table 2 and Supplementary Fig. 2). Changes in cytokines observed during influenza infection might be expected to align with differences after vaccination with live attenuated vaccines. Higher levels of the serum cytokines, including CXCL10 (IP-10), CCL3 (MIP-1α), IL-1β, IL-6, IFN-γ and TNF-α, have been reported in patients infected with influenza^26^. In contrast to infected patients described by Othumpangat et al.^26^ the LAIV post-vaccination sera from subjects in our study had lower IFN-γ and TNF-α levels compared to pre-vaccine levels (Fig. 2, Supplementary Table 2 and Supplementary Fig. 1). This might reflect a difference in the host control of an attenuated virus compared to a wild strain. A large difference between the IIV and LAIV vaccine modalities was observed with IL-1β levels; the levels for both IIV groups were lower, and the levels for both LAIV groups were higher than the pre-vaccination group (0.37 and 0.39 pg/mL for IIV Case and Control groups respectively, 0.72 and 0.67 pg/mL for LAIV Case and Control groups respectively versus 0.52 pg/mL for the pre-vaccination group). IL-1β gene expression in PBMCs was shown to be expressed at lower levels after IIV vaccination in children^23^. However, the IL-1β inter-subject variability was high in our results and these differences were not significant (Supplementary Fig. 2). Some changes in cytokine level are more acute, while others persist for days or weeks. Temporal differences have been described IIV and LAIV vaccinations, particularly for CXCL10^27^, and these described below. These data confirm that cytokine responses differ depending on the vaccine modality and that, even with high inter-subject variably, these differences align with previous descriptions even with serum sampled over a range of days (1–21 days post-vaccination) rather than at a fixed timepoint post-vaccination.

### Post-vaccination cytokine levels correlate with breakthrough infections

**Figure 3:**
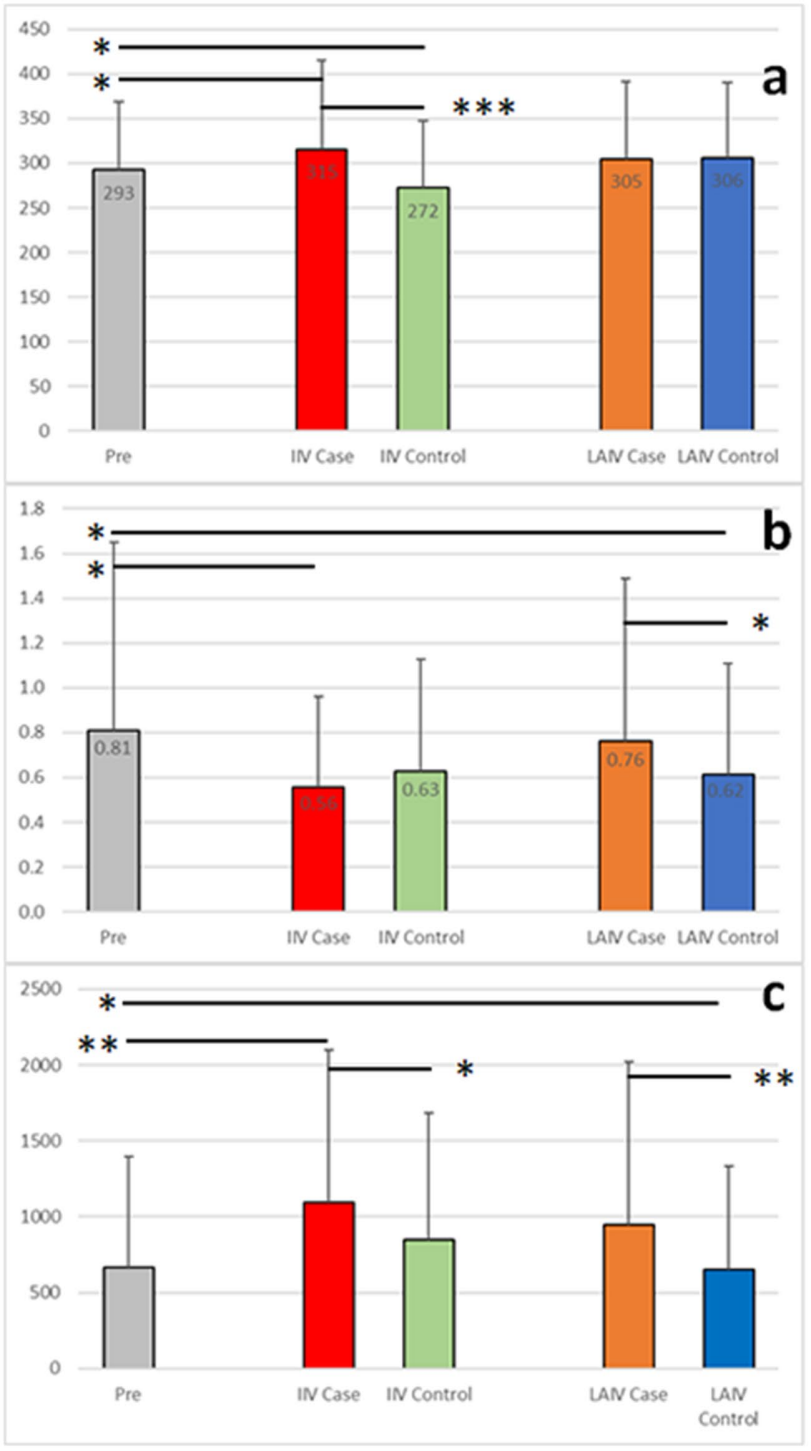
Differences between case and control groups. Serum cytokine measurements (pg/mL) are shown for sCD25 (**a**) and IL-6 (**b**). The sCD25/IL-1β ratios were calculated for each subject and are shown in (**c**). The mean value for each group is shown with standard deviations. *p-value < 0.05; **p-value < 0.01; ***p-value < 0.001.

We specifically undertook these analyses to identify differences in cytokine profiles between subjects who suffered a breakthrough infection and those who did not. Identifying correlates other than specific antibody titers may led to improved vaccines or vaccination strategies^1,13^. Mean sCD25 serum cytokine levels for subjects who received an inactivated vaccine were significantly higher in the IIV Case group (315 pg/mL, p < 0.05) and significantly lower in the IIV Control group (272 pg/mL, p < 0.05) than pre-vaccination levels (293 pg/mL) leading to a greater difference between the IIV Case and Control groups (p < 0.001) (Fig. 3 and Supplementary Table 2). IL-8 levels were significantly lower in the IIV Case group compared to the IIV Control group (21.7 pg/mL versus 31.5 pg/mL, p < 0.05) (Fig. 2). In contrast, neither sCD25 nor IL-8 levels differed significantly between LAIV Case and LAIV Control groups, and post-vaccination sCD25 levels did not differ significantly from pre-vaccine levels in either LAIV post-vaccination group. This indicates that post-vaccination sCD25 and IL-8 levels might be useful in predicting IIV vaccine success.

For subjects receiving LAIV vaccination there is no established correlate of protection, but we observed trends suggesting regulation of interleukins plays an important role. We observed significantly lower levels of IL-6 in the LAIV Control group (0.62 pg/mL) compared to either pre-vaccination (0.81 pg/mL, p < 0.05) or LAIV Case levels (0.76 pg/mL, p < 0.05) (Fig. 3 and Supplementary Table 2). Mean IL-10 levels in the LAIV Control group were also significantly lower than the pre-vaccination levels (0.31 pg/mL versus 0.37 pg/mL, p < 0.05) but, as the mean IL-10 levels for the LAIV Case group (0.35 pg/mL) were slightly lower than the pre-vaccination levels, there was no significant difference between the LAIV Case and Control groups (Supplementary Table 2 and Supplementary Fig. 1). Similarly, CXCL10 (IP-10) levels in the LAIV Case group were significantly higher than the pre-vaccination levels (8.38 pg/mL versus 7.63 pg/mL, p < 0.05) but, as the LAIV Control levels were also higher than the pre-vaccination levels (7.99 pg/mL), there was no significant difference between the two groups (Supplementary Table 2 and Supplementary Fig. 2). Interestingly, like the LAIV Control group, the IIV Case group had lower IL-6 and IL-10 levels. These results indicate that modulation of the interleukin response is important for efficacious LAIV vaccination, and that it differs from the regulation of the immune response required for efficacious IIV vaccination.

We also looked for combinations of cytokine responses associated with breakthrough infections. As described above, there were differences in mean sCD25 levels post-vaccination between the IIV Case and IIV Control groups but not between the LAIV groups. In line with cytokine changes after receipt of inactivated vaccine described by others^23,24^, IL-1β post-vaccination levels are slightly lower than the pre-vaccination levels (0.52 pg/mL) for both the IIV Case group (0.37 pg/mL) and IIV Control group (0.39 pg/mL) but these differences do not reach a level of significance (Supplementary Table 2 and Supplementary Fig. 2). In contrast the mean LAIV post vaccination titers are slightly higher but do not reach a level of significance (LAIV Case, 0.72 pg/mL; LAIV Control, 0.67 pg/mL) (Supplementary Table 2 and Supplementary Fig. 2). When we analyzed the sCD25/IL-1β ratio we observed that values for the IIV Case and LAIV Case groups were significantly higher than the pre-vaccination mean and corresponding Control groups (Fig. 3): The mean sCD25/IL-1β ratio for the IIV Case group is higher than the pre-vaccination ratio (1095 versus 665, p < 0.01) and the IIV Control group (1095 versus 849, p = 0.05), and the mean ratio for the LAIV Case group is higher than the pre-vaccination ratio (945 versus 665, p < 0.05) and the LAIV Control group (945 versus 650, p = 0.01). This indicates that certain combinations of post-vaccination cytokine response may be predictive of either IIV or LAIV vaccine success. However, as described below, some cytokine levels (including IL-1β) change gradually over time which may hinder the usefulness of this approach.

There is high variability in cytokine levels amongst individuals and this is reflected in the broad variance in our data (Figs. 2, 3). We investigated if the variability was affected by the timing of the blood draw. We observed a correlation between some cytokine levels and the number of days post-vaccination that the serum was collected. Most of the significant correlations were observed in LAIV data (see Table 1 below). This observation aligns with another study: Gradual temporal changes in cytokine levels were reported for live attenuated vaccines but not inactivated vaccines^27^.

**Table 1:** Temporal association between cytokine level and number of days post-vaccination. Spearman correlations are shown for results with p-values less than 0.05 for the Case and Control groups according to vaccine modality.

Cytokine	Group	Degrees of freedom	Spearman correlation	p values
BAFF	LAIV Ctrl	138	− 0.199	0.018
CCL2	LAIV Ctrl	135	− 0.285	0.001
CCL3	LAIV Ctrl	121	− 0.281	0.002
SCD25	LAIV Ctrl	134	− 0.173	0.044
IFN-γ	LAIV Ctrl	143	− 0.185	0.026
IL-1β	LAIV Ctrl	120	− 0.206	0.023
IL-1β	LAIV case	119	− 0.287	0.001
CXCL10 (IP-10)	LAIV case	128	0.177	0.044
TNF-α	LAIV case	124	− 0.179	0.045
IL-6	IIV case	82	− 0.217	0.047

Clinical trials that follow a small number of subjects over a series of hours or days post-vaccination have been published^21,22,27^. While these studies are valuable in assessing changes in immune function after vaccination, they are not directly linked to vaccine success or breakthrough infections. The work by Weiner et al.^27^ highlights the acute increase in CXCL10 (IP-10) serum levels during the first 2 days after receipt of inactivated vaccines in contrast to the more gradual increase over 7 days observed after receipt of live attenuated vaccines. Similarly, we observed a correlation between the number of days post-vaccination that serum was collected and cytokine level. Correlations were observed for several cytokines in the LAIV groups but for only one cytokine in the IIV group (cytokines with Spearman correlations p values < 0.05 are provided in Table 1). The more significant correlations were negative correlations with the CCL2 and CCL3 in the LAIV Control group and IL-1β in the LAIV Case group. The trends were similar for CCL2 and CCL3 in the LAIV Case group but did not reach significance (− 0.169, p = 0.054, df = 129 and − 0.166, p = 0.074, df = 120 respectively). We observed a positive correlation between the day of blood draw and CXCL10 (IP-10) serum levels for the LAIV Case subjects (0.177, p = 0.044, df = 128) and a non-significant negative correlation for the LAIV Control group (− 0.148, p = 0.088, df = 131). We observed a negative correlation between the day of blood draw and BAFF (p < 0.05) for the LAIV Control group but not the LAIV Case group or either IIV group. These correlations indicate that temporal changes in post-vaccination cytokine levels tend to be associated with LAIV rather than IIV vaccinations.

### Differences between male and female post-vaccination responses

Males and females are known to respond differently to influenza infection and vaccination^28,29^. We performed subgroup analyses to determine if there were differences in cytokine levels between the groups that might provide insight into these differences. The majority of serum samples available for analysis were from male subjects with only 13% (79/599) from females. There were seven cytokines for which the mean levels of both the pre- and post-vaccination sera differed significantly (p-values < 0.05) between male and female samples: TNF-α, IFN-γ, IL-8, IL-10, CCL2, CCL3 and sCD25. The pre-vaccination levels for all of these cytokines except IL-10 were significantly lower in the female datasets compared to the male (Supplementary Fig. 3). Even with different baseline cytokine levels several of the patterns remained for both sexes. Different patterns were observed for IFN-γ, IL-8, CCL2 and CCL3.

The mean pre-vaccination IFN-γ levels for females (0.92 pg/mL) was much lower than that for males (2.17 pg/mL). All post-vaccination mean levels were higher than the mean pre-vaccination level for females and lower for males. The trend of higher IFN-γ levels in the LAIV groups compared to the IIV groups persisted for both sexes with no significant difference between the respective Case and Control groups. The pre-vaccination IL-8 levels were lower in females but lower post-vaccination IL-8 levels were still apparent for the Control groups for when both sexes were analyzed separately (males, p < 0.001; females, p < 0.05). Interestingly, the mean IL-8 levels for the IIV Case groups were significantly lower (p < 0.05) for females and higher (p < 0.001) for males than the respective IIV Control groups. In addition, the LAIV Control levels were significantly higher than LAIV Case levels (p < 0.01) for females.

Although pre-vaccination CCL2 levels were lower in females than males (132 pg/mL versus 188 pg/mL), the post-vaccination levels for both IIV Case and Control groups were similar among the sexes (175–192 pg/mL). This resulted in significantly higher relative post-vaccine titers for the female IIV groups but not for the males (or combined datasets). There were no significant differences between the pre- and post-vaccination CCL3 levels for the combined data or when males were analyzed separately. However, higher CCL3 levels for the female LAIV Control group and lower levels for the IIV Control group resulted in significant differences between Case and Control groups for both IIV and LAIV (p < 0.05 and p < 0.01 respectively).

A similar pattern was observed with TNF-α levels for both men and women even though the absolute levels were lower for females. Post-vaccination TNF-α levels were lower than pre-vaccination averages for both IIV Case and Control groups for both sexes, but the significance was lost in the smaller female dataset. The mean TNF-α levels for females in the LAIV Control group were significantly higher (p < 0.05) than the LAIV Case groups. For IL-10 the mean pre-vaccination levels were higher in females, but similar differences were observed for IIV groups from pre-vaccination levels; significantly lower levels in the IIV Case groups and similar levels for IIV Control groups.

Although female pre-vaccination sCD25 levels were lower than male (208 pg/mL versus 301 pg/mL) the trend for higher levels in the IIV Case groups compared to IIV Control observed in the combined dataset persisted in both subgroups but was not significant for the female dataset. Of note, while the sCD25/IL-1β ratios were higher for the IIV and LAIV Case groups than Control groups for the total dataset, this pattern was lost for the male IIV groups and female LAIV groups.

### Analysis of outliers

**Figure 4:**
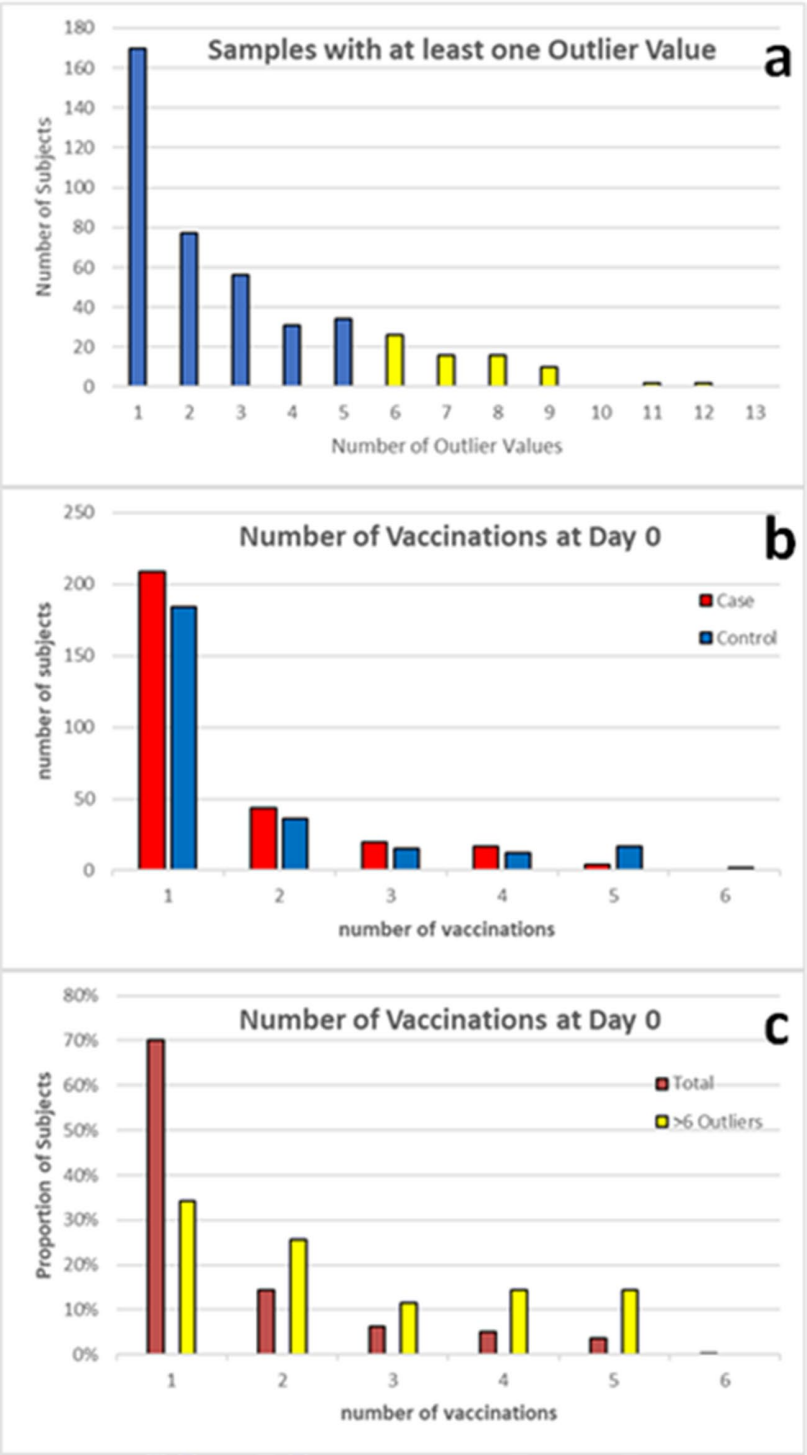
Relationship between outlying cytokine values and number of vaccinations. The number of samples with one or more outlying value were identified (**a**). The Case and Control groups are compared based on the number of subjects receiving multiple vaccinations (**b**). The proportion of total subjects receiving multiple vaccination is compared with the portion of subjects with sera presenting 6 or more outlying cytokine measurements (yellow bars in **a**,**c**).

It might be expected that if a sample had an outlying value for one cytokine, then other cytokines from that sample might also skew higher or lower from the rest of the group if the subject had an underlying medical condition at that time. We were interested if specific samples were broadly responsible for wide variances observed. We found that 75% of samples had an outlying value for at least one of the 15 cytokine measurements (Fig. 4) and of those 16.8% had six or more cytokine measurements that were outliers. These outliers are spread among the different groups: Of the 76 post-vaccination samples with six or more outlying values 36 were from Case sera and 40 from Control sera; 36 from IIV sera and 40 from LAIV sera. This indicates that the presence of outlying values was not associated with the type of influenza vaccine, nor breakthrough infections.

We performed additional ad hoc assessments of the samples with multiple outlying cytokine levels. Of the 87 samples with outlying TNF-α values, 40 (12 pre-vaccination and 28 post-vaccination samples) also had outlying values for IL-1β, IL-6 and IL-8. Like the samples with six or more outliers described above, these were spread throughout the different groups and included 4 paired samples and 7 matched samples. This suggests that outlying levels for these cytokines may persist in some subjects and that the matching criteria may have paired subjects with common characteristics associated with outlying values.

The distribution curves for Control and Case subjects were similar with regard to the number of vaccines concomitantly administered (Fig. 4). We checked to see if similar vaccines were administered to subjects with outlying TNF-α, IL-1β, IL-6 and IL-8 values. Of the 28 subjects with post-vaccination samples, seven had records indicating receipt of only the influenza vaccine, and all but three of the remainder had anthrax and/or tetanus vaccines administered concomitantly with the influenza vaccine. Anthrax vaccination has been shown to increase TNF-α levels^30^ and tetanus vaccination increases IFN-γ levels^31^. For all sera samples there were 118 records (20% of all sera samples) indicating anthrax vaccination during the 30 days prior to the blood draw, and 14/118 had six or more cytokine measurements that were outliers. Similarly, there were 124 records of tetanus vaccination prior to blood draws representing 21% of all sera samples and 16/124 were associated with multiple outliers (including 6 that had also received anthrax vaccinations within the same timeframe). Anthrax and Tetanus vaccinations are known to lead to changes in cytokine levels^30–32^. From the limited medical data included with the serum samples for this study we observed that the distribution of subjects presenting > 6 outlier values differed from the distribution of all subjects according to the number of concomitant vaccines (Fig. 4). This indicates that receipt of multiple vaccine may be associated with a range of cytokine measurements beyond the normal range in the DoD population.

### Effects of concomitant vaccination

We performed subgroup analyses based on receipt of any other vaccine 30 days prior to influenza vaccination or between the influenza vaccination and the post-vaccination blood draw (Supplementary Fig. 4). The subgroups were fairly evenly split with 47% of the post-vaccination samples from subjects without additional vaccines and 53% from subjects receiving concomitant vaccines. Within those groups the splits between Case and Control groups for each vaccine modality were also fairly even (from 46 to 54%).

**Figure 5:**
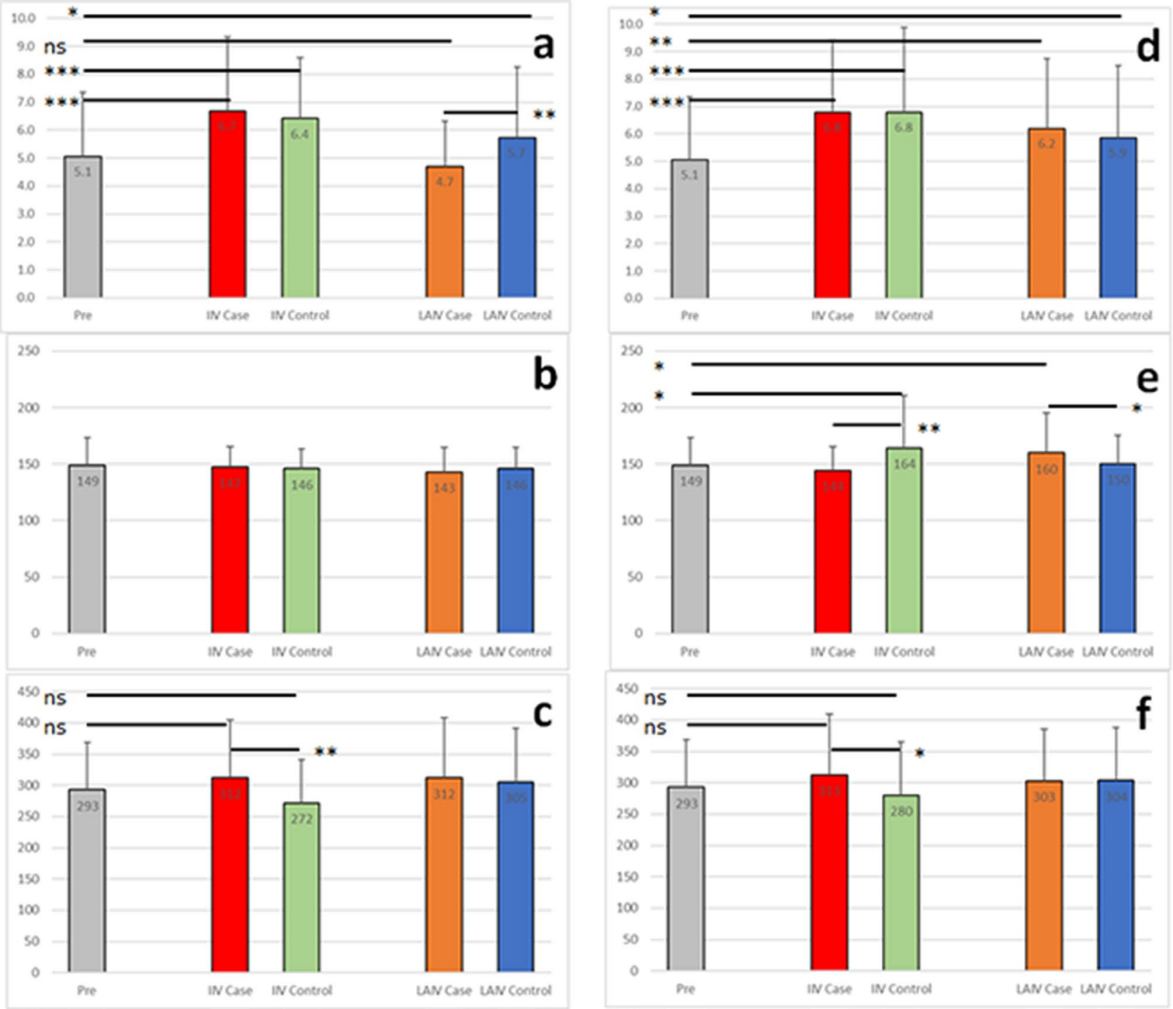
Antibody and cytokine values with (**a**–**c**) or without (**d**–**f**) concomitant vaccinations. Hemagglutination inhibition titers (log2) toward the A/California/07/2009 strain are shown in panels (**a**) and (**d**). Serum cytokine measurements (pg/mL) are shown for CCL3 (**b**,**e**) and sCD25 (**c**,**f**). The mean value for each group is shown with standard deviations. *p-value < 0.05; **p-value < 0.01; ***p-value < 0.001.

Differences in antibody titers were observed between the groups with or without additional vaccinations. There were no significant differences in HI titers before and after vaccination for the LAIV groups for the A/Brisbane/59/2007 vaccine strain recommended prior to the pandemic (Supplementary Fig. 4). There were slightly higher titers in the IIV Control groups (p < 0.05) with or without additional vaccines. The IIV Case group with concomitant vaccination had a significantly higher post-vaccination titer (p < 0.001) that was not observed for the IIV Case group receiving IIV alone. Hemagglutination inhibition titers for the A/California/07/2009 strain recommended in the first year of the H1N1 pandemic were significantly higher (p < 0.001) for both IIV Case and Control groups regardless of additional vaccine receipt or not (Fig. 5). Interestingly, there was a difference in A/California/07/2009 titers in the LAIV Case groups associated with receipt of additional vaccines. There were significantly higher titers for the LAIV Case (p < 0.01) and Control (p < 0.05) groups when no additional vaccine was administered but titers were lower in the LAIV Case group receiving concomitant vaccines that resulted in a significant difference between these LAIV Case and Control groups (4.7 versus 5.7, p < 0.01). These data indicate that concomitant vaccination may have contributed to a back boost for A/Brisbane/59/2007 HI titers in the IIV Case group and a reduced A/California/07/2009 antibody response for the LAIV Case group. But these differences in antibody cannot be the sole factor in subsequent breakthrough infections because similar numbers of breakthrough infections were observed in the groups not receiving additional vaccines where there were no differences in mean antibody titer between those Case and Control groups.

Concomitant vaccination alters the cytokine responses, especially for subjects receiving LAIV. Analysis of the subgroup not receiving additional vaccines revealed significant differences in post-vaccination CCL2, CCL3 and CXCL10 levels (p < 0.05) that resulted in differences between the LAIV Case and Control groups (p < 0.05) not observed in the subgroup receiving additional vaccines (Fig. 5, Supplementary Tables 3 and 4, and Supplementary Fig. 4). CCL2 and CCL3 are induced by inflammation and CXCL10 is an interferon protein so we next compared the pro-inflammatory cytokine and IFN-γ levels. There was no difference in IFN-γ levels between the LAIV Case and Control groups with or without concomitant vaccination. However, there was a significant difference in the levels of the pro-inflammatory cytokines. IL-1β and IL-8 levels were significantly higher (p < 0.01) when the LAIV Case and Control groups without concomitant vaccines were compared (Supplementary Table 3 and Supplementary Fig. 4). The same pattern was observed for IL-6 and IL-10 (p < 0.001 and p < 0.05 respectively). In contrast, in the subgroup receiving concomitant vaccines the trend was lost (IL-6 and IL-10) or reversed with IL-1β and IL-8 levels significantly higher (p < 0.001) in the LAIV Control groups compared to the LAIV Case groups (Supplementary Table 4 and Supplementary Fig. 4). These data demonstrate that additional vaccines affect post-vaccination cytokine levels compared to LAIV administration alone. As there were similar numbers of breakthrough infections among the two subgroups (with or without concomitant vaccines) these differences in post-vaccination cytokine levels by themselves cannot account for subsequent susceptibility to breakthrough infection.

Distinctions between IIV Case and IIV Control groups remained apparent regardless of concomitant vaccination for most cytokine levels. In the subgroup analysis the pattern remained the same for sCD25 levels although the significance for the difference between the IIV Case and IIV Controls was not as strong as seen with the complete dataset (p < 0.01 for concomitant vaccines and p < 0.05 for no additional vaccines compared to p < 0.001 for the complete group) (Supplementary Tables 3 and 4, and Supplementary Fig. 4). The trend for higher sCD14 levels in the IIV Case groups compared to the IIV Control groups remained but the significance was reduced (p < 0.05 for IIV alone, p = 0.17 in the concomitant subgroup) (Supplementary Table 4 and Supplementary Fig. 4). The pattern remained the same with lower IFN-γ levels observed in the IIV Case and Control groups with or without receipt of additional vaccines (Supplementary Tables 3 and 4, and Supplementary Fig. 4). These data indicate that, although concomitant vaccination affects post-vaccination cytokine levels, post-vaccination sCD25 levels are indicative of subsequent susceptibility to breakthrough infections.

## Discussion

In this study we have identified serum cytokines levels that changed after vaccination and describe differences in cytokine levels in subjects that subsequently suffered breakthrough infections. Mean cytokine levels differed from pre-vaccination levels for both IIV and LAIV groups for several of the assayed cytokines. There was a trend for reduced cytokine levels after either vaccine modality (IL-6, IL-8, IL-10, IFN-γ and TNF-α) while some, such as IL-1β and CXCL10, increased after LAIV vaccination but not after IIV vaccination. We also report differences between the Case and Control groups demonstrating the important role cytokines have in successful vaccination. Large variances in cytokines levels may be indicative of different immune challenges among subjects at the time of vaccination. In our cohorts, receipt of additional vaccines masked some differences between Case and Control groups, especially for LAIV recipients.

The humoral response is characterized by the formation of antibodies which play an important role in protection from disease. Antibody titers are used as a correlate of protection for IIV and a connection between antibody titers and BAFF cytokine levels has previously been demonstrated. Hill et al.^33^ observed a negative correlation between antibody and BAFF levels after vaccination an adult population linking B cell stimulation to antibody production. We also observed a decrease in post-vaccination BAFF levels and higher antibody titers for the IIV groups but not the LAIV groups confirming this association for inactivated influenza vaccines (Supplementary Fig. 1). However, the IIV Case and Control groups had the same mean post-vaccination antibody titers indicating that something beyond the antibody titer contributes to vaccine efficacy. Our investigation of post-vaccine cytokine levels indicates that control of the immune response, inflammation in particular, plays a significant role in vaccine induced protection.

Beyond antibody production, the parts of the immune response needed for an efficacious vaccine are less well characterized^34^. Temporary increases in IFN-γ and CXCL10 have been observed after mRNA vaccination^35^. We also observed higher CXCL10 levels after vaccination and other responses specific to the type of vaccine received. Cytokine levels have been measured before and after LAIV vaccination by others^36^. Like Stein et al.^36^ we did not observe significant differences in the mean pre- and post-vaccination for IFN-γ, CCL2 (MCP-1) or CCL3 (MIP-1α) levels after LAIV vaccination. Both TNF-α and IFN-γ levels trended lower after LAIV vaccination in line with the results from Stein et al.^36^. TNF-α and IFN-γ levels were significantly lower after IIV vaccination indicating more robust modulation of that part of the immune response with IIV administration (Fig. 2, Supplementary Table 2 and Supplementary Fig. 1). This trend persisted with IIV subgroups receiving additional vaccines (Supplementary Fig. 4) but not for IFN-γ in the female subgroup with lower pre-vaccine levels (Supplementary Fig. 3). Higher CXCL10 levels after both IIV and LAIV influenza vaccination have previously been reported^6,22,27,36^. Weiner et al.^27^ showed that the change in CXCL10 levels differ between IIV and LAIV, with an acute response for IIV and a more sustained change for LAIV. This is also reflected in our data with higher mean CXCL10 levels after LAIV compared to IIV (Supplementary Table 2 and Supplementary Fig. 2). Interestingly, a positive temporal correlation between CXCL10 levels and timing of the blood draw post-vaccination was observed in the LAIV Case group but not the LAIV Control group (Table 1). This suggests that a return to normal levels was abrogated in the Case group. Changes in gene expression in PBMCs after receipt of IIV or LAIV in children also included upregulation of CXCL10 and downregulation of IL-6 after LAIV (Ref.^23^, Supplementary Table 3) as was observed in the young adult population studied here (Supplementary Fig. 1 and Fig. 3). In another study, more than 10% of samples from an adult population assessed had CXCL10 or IL-6 levels below the limit of detection so statistical analyses were not performed preventing a comparison^36^. Several cytokines (including IL-6, IL-8, IL-10 IL-1β, BAFF, IFN-γ and TNF-α) have been shown to drop below baseline levels after receipt of inactivated influenza vaccines^20–22,24^. We also observed decreases in these cytokine levels with the IIV cohort (Figs. 1 and 2, Supplementary Table 2 and Supplementary Fig. 1). The change in these cytokine levels after vaccination is the opposite of the recall response observed when peripheral blood mononuclear cells (PBMCs) are stimulated^37^ suggesting that cytokines secreted from other cells play an important role.

Our analysis of post-vaccination cytokine levels between subjects who succumbed to breakthrough infections and those who did not indicates the difference could be related to the immune status at the time of vaccination. Altered cytokine levels have been observed for different diseases or chronic conditions. Higher levels of circulating IL-6 and IL-1β have been observed in patients with chronic pulmonary sequelae, and higher circulating IL-1β and TNF-α levels are involved in signaling to repair damaged lung tissue^38^. Several cytokine levels trended higher in the LAIV groups than the IIV groups suggesting greater systemic inflammation after LAIV vaccination (IL-1β, IL-6, IL-8, IL-10 and CXCL10: Fig. 2, Supplementary Table 2, and Supplementary Figs. 1 and 2). We also observed significantly higher levels of IL-6 and IL-1β in LAIV Case subgroup without additional vaccines compared to the LAIV Control subgroup. This suggests a difference in control of the attenuated virus that subsequently affected vaccine efficacy (Supplementary Tables 3 and 4, and Supplementary Fig. 4). This difference remained significant for IL-6 but not IL-1β when subjects with concomitant vaccines were included (Fig. 3). For the IIV recipient group lower levels of interleukins 6, 8 and 10 were observed in the Case group compared to the Control group with the difference reaching significance for IL-8 (Supplementary Table 2 and Supplementary Fig. 1). In addition, expression of the alpha-chain of the interleukin 2 receptor (sCD25) differed significantly between the IIV Case and Control groups (Fig. 2 and Supplementary Table 2). This indicates that controlling inflammation and the activation of T-cells after vaccination plays an important role in establishing an effective response to vaccination.

Individual host attributes, such as prior exposures or vaccinations, may affect the response to vaccination. However, there is a limited body of work describing how these differences affect vaccine efficacy. Many prior analyses have focused on the antibody response to repeat influenza vaccinations^39–41^. Vaccination itself alters the immune landscape at least temporally^6,22,27^, and possibly for months or years^42,43^. Receipt of additional vaccines within the same timeframe as the influenza vaccine was a prominent confounder in our analysis. We observed that subjects receiving concomitant anthrax or tetanus vaccines were more likely to have multiple cytokine values that were outliers signifying a skewed immune response. Differences were more notable for LAIV than IIV. For example, the distinction between LAIV Case and Control groups for IL-6 and CXCL10 was reduced or reversed when subjects receiving additional vaccines were assessed separately (Supplementary Tables 3 and 4, and Supplementary Fig. 4). Elevated sera CXCL10 levels have previously been associated with some adverse events (large local reactions) but not others (systemic reactions) after anthrax vaccination^44^. Much lower levels of IL-6 were observed in the IIV and LAIV Case subgroups with additional vaccination compared to the IIV and LAIV subgroups with no concomitant vaccines in line with the decrease in IL-6 observed in mice vaccinated with anthrax^45^. The immune stimulatory power of anthrax toxin has been harnessed as a potential adjuvant to broaden the specificity of influenza vaccines^46^. It has been suggested that receiving multiple concomitant vaccines, or certain combinations of vaccines, could be a contributing factor in adverse outcomes^47^. However, we observed breakthrough infections in both subgroups (with or without concomitant vaccines) suggesting more complex reasons for breakthrough infections. It appears from our work that there is a correlation between breakthrough infections and cytokine levels after LAIV that is affected by concomitant vaccination. There is evidence of non-specific effects, including protection from respiratory disease, from other live vaccines in children^42,48^. This would suggest that some aspect of the immune response to the live vaccines is beneficial irrespective of the antigen content. We hypothesize that additional concomitant vaccines altering post-vaccination immune status may mute this effect.

Our results suggest that vaccine efficacy is better with an optimal post-vaccination cytokine response but there is a lack of information connecting these parameters. An epidemiological approach comparing vaccine efficacy in populations that are more obese with populations that are less obese might collaborate some of these findings. Adjustments are sometimes made to factor in obesity when making comparisons among countries^49^. Differing obesity and metabolic phenotypes are associated with different post-vaccination cytokine levels even though antibody response is the same^50^. Despite robust antibody responses some studies indicate vaccinated obese suffer more infections^51^. This is not the case for all types of vaccines as higher morbidity and mortality from SARS-CoV-2 was not observed for obese subjects vaccinated against COVID-19^52^. Amending the immune response to vaccination by using cytokines as adjuvants has been investigated but toxicity and nonspecific effects on different T-cell populations present major challenges with this approach^53,54^. Other approaches such as diet modification to moderate the inflammatory response could have role in facilitating an optimal response to vaccination. Supplements that have been shown to prevent influenza infection in clinical trials are thought to achieve this effect by moderating the inflammatory response^55^. An awareness of links between pre-vaccination immune status and possible post-vaccination outcomes (in addition to antibody response) may be informative in selecting the optimal vaccine (or adjuvant), and optimal timing of vaccination, for individual patients.

There are several limitations to our study. The population was limited to a young adult population which doesn’t bear the brunt of annual influenza morbidity and mortality. We do not know if our observations will extend to immune responses in older and younger populations where prevention of disease has more impact. The study population is a highly vaccinated one and the receipt of additional vaccines ablated several differences between Case and Control groups. Although the majority of blood draws in this population are routine, we cannot discount the possibility that the blood draws could have been initiated in response to a medical event that may have also altered cytokine levels. The timing of the blood draws with respect to the time of vaccination is not fixed as in traditional trial design and this hampers comparisons. We performed our analysis on sera samples that had been frozen and thawed at least twice. While we were able to apply the same conditions to all samples processed in our laboratory, we do not know if prior differences in sample treatment could have contributed to cytokine stability or degradation. The timing of sample collection post-vaccination covered a range of 1–21 days. Although our results are concordant with other analyses, the detection of temporal associations requires further investigation. The study was performed using samples for the 2009/10 season, the first winter season during which the 2009 pdmH1N1 strain was predominant and the likely cause of influenza infections. Cytokine responses to vaccines for an antigenically shifted strain may differ from responses to vaccines updated for antigenically drifted strains to which the body could have prior experience. Even with these limitations we provide evidence of differences in post-vaccination cytokine levels that correlate with breakthrough infections. Our identification of differences in cytokine levels that correlate with vaccine breakthrough infections for two different influenza vaccine modalities opens up new avenues for investigating and understanding how vaccine efficacy is achieved.

## Materials and methods

Sera were obtained from the Department of Defense Serum Repository. The study was approved by the Defense Health Agency Office of Research Protections. All methods were performed in accordance with relevant guidelines and regulations including the de-identification of samples before release.

The study population consisted of service members with documented influenza vaccination between October 1st and December 15th 2009 (CVX codes 111, 140, 141, 015, 088, 123, 125, 126, 127 and 128). The first criterion is that samples from the test group were identified as influenza cases; any subject having an inpatient or outpatient medical encounter with an influenza like illness (ILI) diagnosis. These include ICD codes that indicate an infection is caused by influenza but do not state that influenza infection was confirmed (ICD-9: 079.99, 382.9, 460, 461.9, 465.8, 466.0, 486, 488.0, 488.8, 488.81,488.02, 488.82, 488.09, 488.1, 488.19, 488.89, 487.0, 487.1, 488.12, 487.8, 490, 786.2, 780.6, 780.60; ICD-10: J09–J11). However, there are strong correlations between some codes and confirmed influenza^56^ and some codes have been used in other studies to assess the impact of vaccination on pdmH1N1 infection^57^. Subjects diagnosed with influenza within 14 days of vaccination were excluded because of the possibility that they had already been infected before protection from the vaccination was achieved. The next criterion was that a serum sample was taken 1–21 days post-vaccination. Immediately after vaccination there are changes in the cytokine and chemokine levels^22^. We are interested in determining if there is a correlation between these signals and vaccine breakthrough infections. If available, a pre-vaccination serum sample within the six months prior to vaccination was included.

The samples for the control group were matched to each test subject based on the following criteria: Type of vaccination (2009/10 seasonal or pdmH1N1); timing of blood draw relative to the vaccination (same day ± 1); any second influenza vaccine received that season (2009/10 seasonal or pdmH1N1); no evidence of an influenza like illness during the entire influenza season (no inpatient or outpatient medical encounter with ILI diagnosis (ICD-9-CM Codes: 460.xx, 486.xx, 488.xx, 490.xx, 079.99, 382.9, 461.9, 465.8, 465.9, 466.0, 487.0, 487.1, 487.8, 786.2, 780.60; ICD-10-CM Codes: B97.89, H66.9, H66.90, H66.91, H66.92, H66.93, J00, J01.9, J01.90, J06.9, J09, J09X, J09.X1, J09.X2, J09.X3, J09.X9 ,J10, J10.0, J10.00, J10.01, J10.08, J10.1, J10.2, J10.8, J10.81, J10.82, J10.83, J10.89, J11, J11.0, J11.00, J11.08, J11.1, J11.2, J11.8, J11.81, J11.82, J11.83, J11.89, J12.89, J12.9, J18, J18.1, J18.8, J18.9, J20.9, J40, R05, R50.9); and other descriptive demographic attributes like the test case sample (sex, age, race) where possible. The date and CVX codes for any concurrent vaccines received from 30 days before the influenza vaccination and demographic information (age, sex and race) were included. The exclusion criteria applied to the study population were immunosuppressed individuals (279.3), pregnant individuals (V22.0), and those with any serious active disease [e.g. cancer (140–174), congestive heart failure (428), chronic obstructive pulmonary disease (496), autoimmune disease 279.49, asthma (493.90)].

Cytokines were measured using 96-well multiplex plates from R&D Systems (Minneapolis, MN, USA) in a Luminex FlexMap 3D multiplex plate reader. The cytokines assayed were C-Reactive Protein (CRP), sCD14, BAFF/BLyS, CCL2/MCP-1, CCL3/MIP-1α, sCD163, sCD25/IL-2R alpha, CXCL10/IP-10, sIL-6R alpha, IFN-γ, IL-1 beta, IL-6, IL-8/CXCL8, IL-10 and TNF-alpha). The serum samples were thawed and aliquoted at the appropriate dilution for the Luminex assay. All plates included standards and a control sample.

Mean cytokine levels for the different groups were compared: The post-vaccination cytokine levels for the case and control groups for each vaccine modality (IIV or LAIV) were compared to the pre-vaccine group cytokine levels to identify changes in response to vaccination; the case and control groups were compared to identify differences that correlated with breakthrough infections; and the paired samples from individual subjects were visualized to assess if the individual patterns were comparable to the group data. The interquartile range (IQR, 25th–75th percentile) for the results of each cytokine for each group were determined and outliers (beyond the median ± 1.5 × IQR) were removed. Student T-tests were used to assess the significance of differences between groups.

Hemagglutination inhibition (HI) assays were performed as previously described^40^. Briefly, one volume of serum was treated with three volumes of receptor destroying enzyme (RDE; Accurate Chemical, Westbury, NJ, USA) at 37 °C overnight. The RDE was inactivated by heat treatment at 56 °C for 30 min followed by the addition of six volumes of PBS to reach a final dilution of 1:10. Two-fold dilutions of the sera samples were made across a 96-well plate leaving 25 µl in the first 11 columns. PBS was added to the 12th column as a no sera control. An equal volume of virus titrated to 4 hemagglutinin units was added to the first 11 columns and the plates were incubated at room temperature to allow antibodies to bind virus. Then turkey red blood cells (RBCs, 0.5%) were added to all wells and the plates incubated for 1 h at room temperature to allow time for the RBCs to pellet or agglutinate if insufficient virus remained sequestered by antibodies. The HI titer is reported as the last dilution of sera that completely inhibited agglutination.

## Supplementary Information

Supplementary Figures.

Supplementary Tables.

## Data Availability

The datasets used and/or analysed during the current study available from the corresponding author on reasonable request.
