# Supplementary material for: Post-vaccination serum cytokines levels correlate with breakthrough influenza infections
Source: Sci Rep. 2023 Jan 20;13:1174. doi: 10.1038/s41598-023-28295-8 (PMC9857916; doi:10.1038/s41598-023-28295-8)
Supplement: Supplementary file 1 — Supplementary Figures. [file 41598_2023_28295_MOESM1_ESM.pdf]

Supplementary Figure 1

Supplementary Figure 1. Lower cytokine levels observed after either vaccination. Hemagglutination inhibition titers (log2) toward the A/Brisbane/59/2007 strain are shown to indicate the slight increase (back-boost) toward a previous season antigen after vaccination.
Serum cytokine measurements (pg/mL) are shown for TNF-α, BAFF, IL-6 IL-8 and IL-10. The mean value for each group is shown with standard deviations. *, p-value <0.05; **, p-value < 0.01; ***, p-value < 0.001.

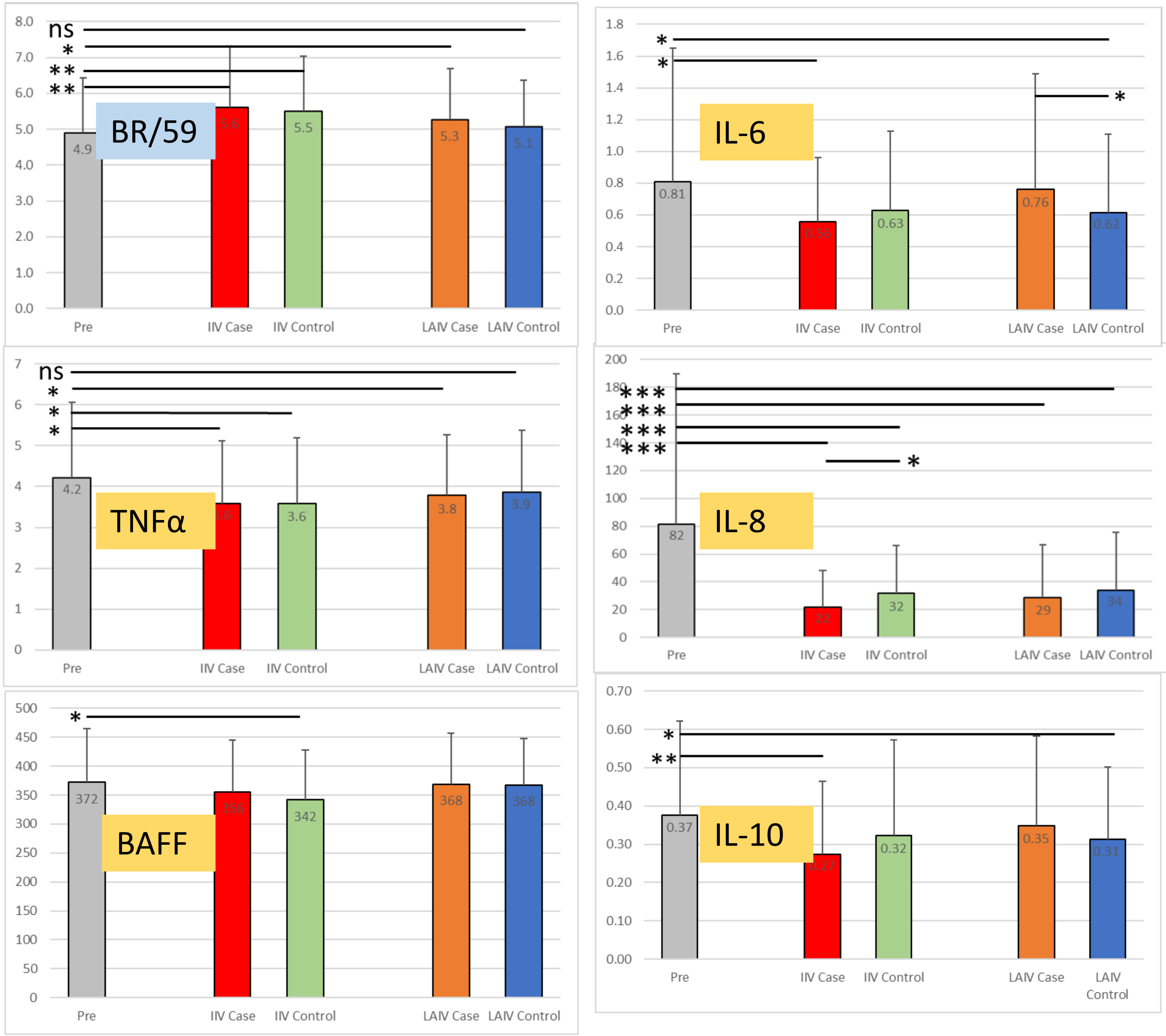

Supplementary Figure 2

Supplementary Figure 2. Changes in cytokine levels that differ depending on vaccine modality.
Serum cytokine measurements (pg/mL) are shown for IL-1β and CXCL10. The mean value for each group is shown with standard deviations. *, p-value <0.05.

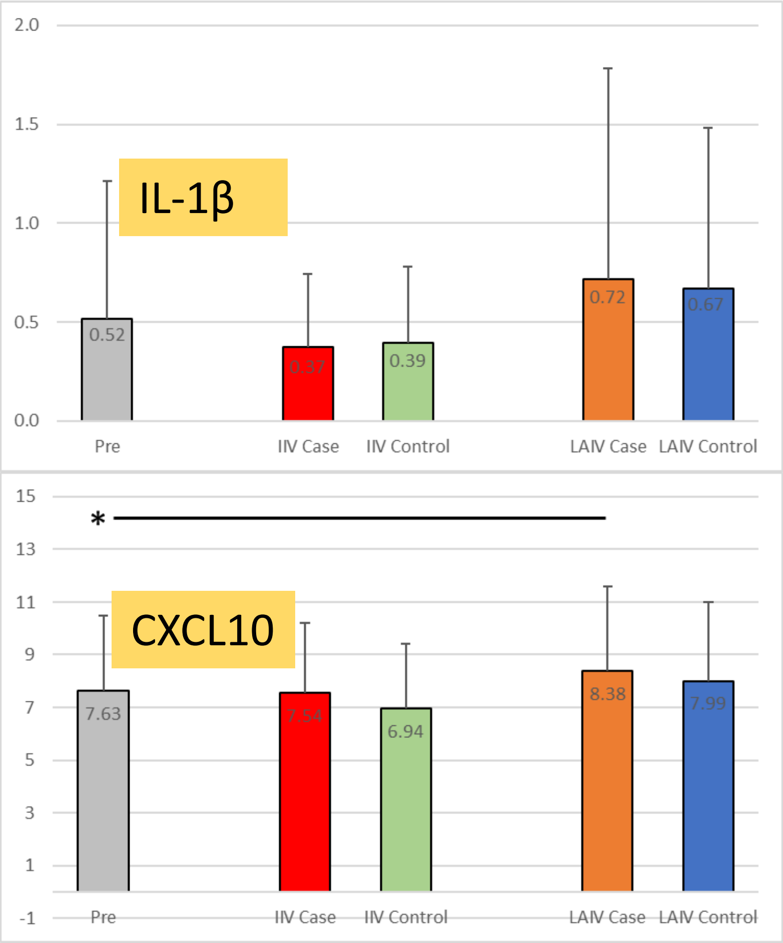

Supplementary Figure 3

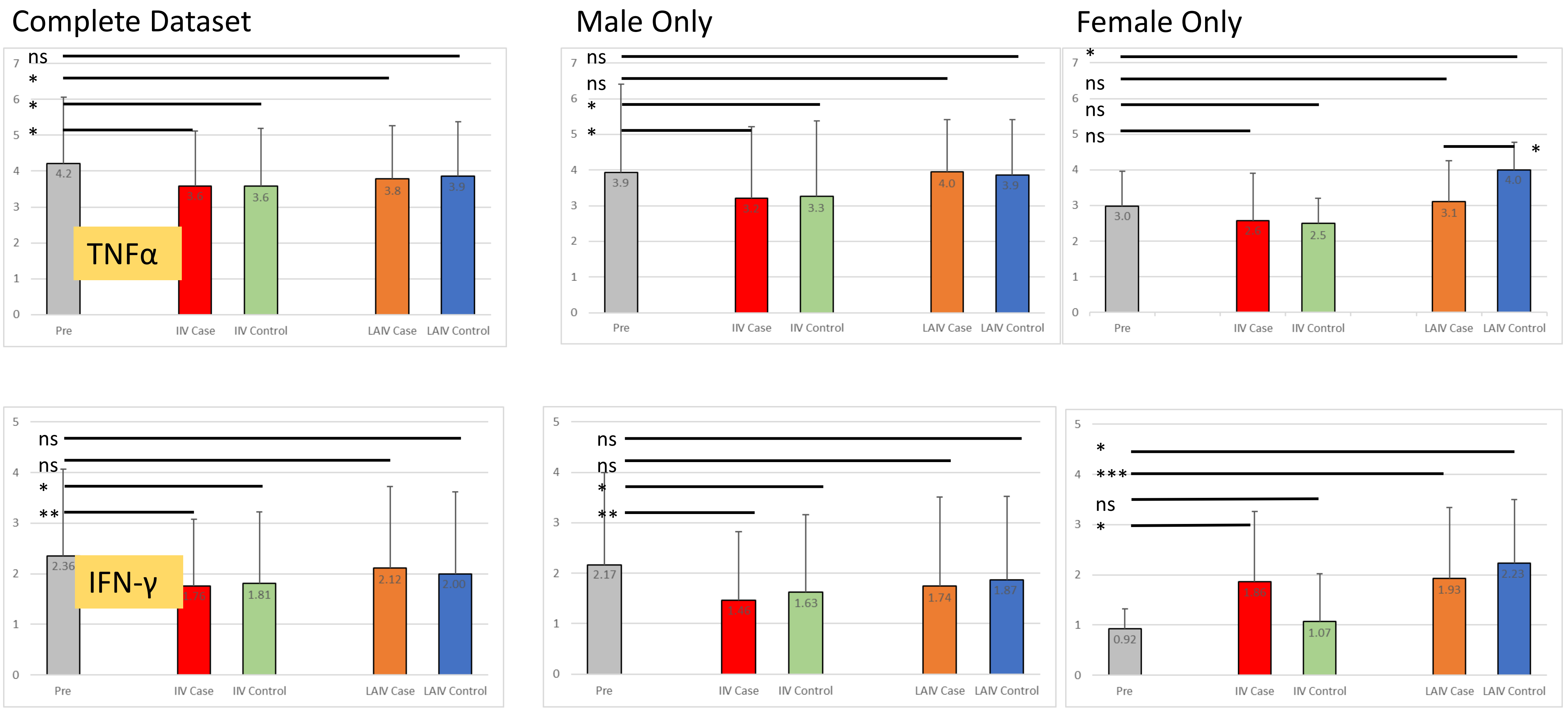

Supplementary Figure 3. Comparison of subgroups according to sex with complete dataset. Serum cytokine measurements (pg/mL) are shown for TNFα and IFN-γ. The mean value for each group is shown with standard deviations. ns, not significant; *, p-value <0.05; **, p-value < 0.01; ***, p-value < 0.001.

Supplementary Figure 3 continued

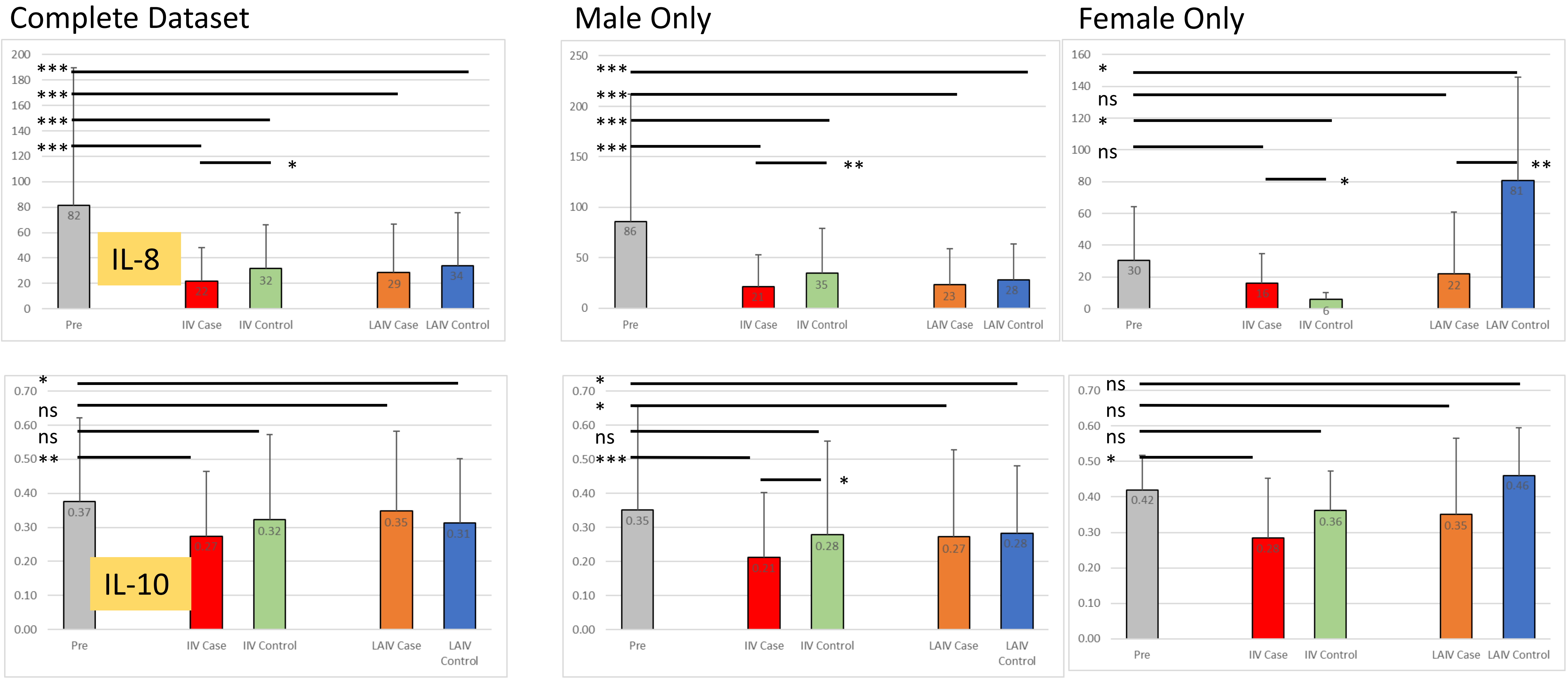

Supplementary Figure 3 continued. Comparison of subgroups according to sex with complete dataset. Serum cytokine measurements (pg/mL) are shown for IL-8 and IL-10. The mean value for each group is shown with standard deviations. ns, not significant; *, p-value <0.05; **, p-value < 0.01; ***, p-value < 0.001.

Supplementary Figure 3 continued

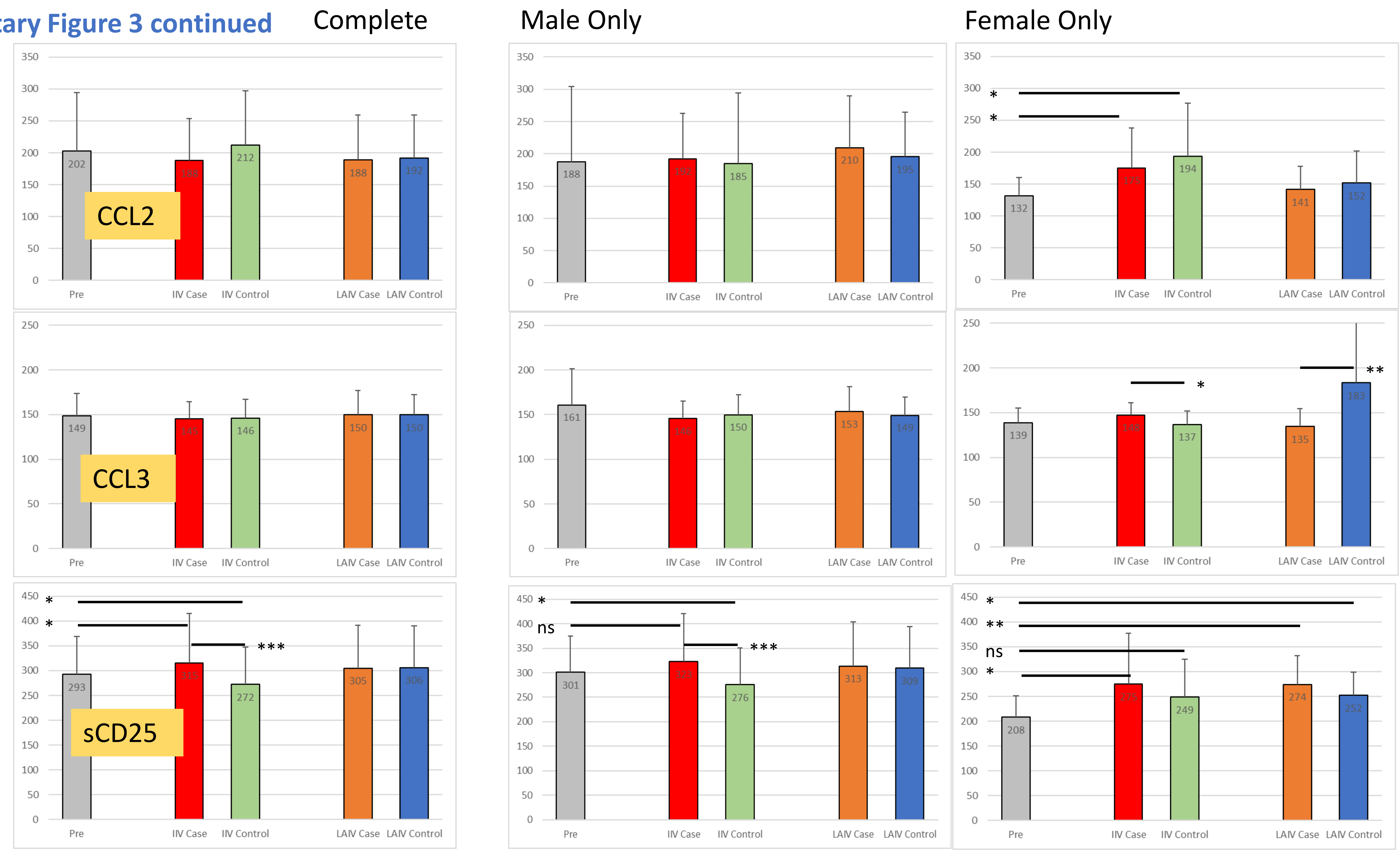

Supplementary Figure 3 continued

Complete | Male only | Female only

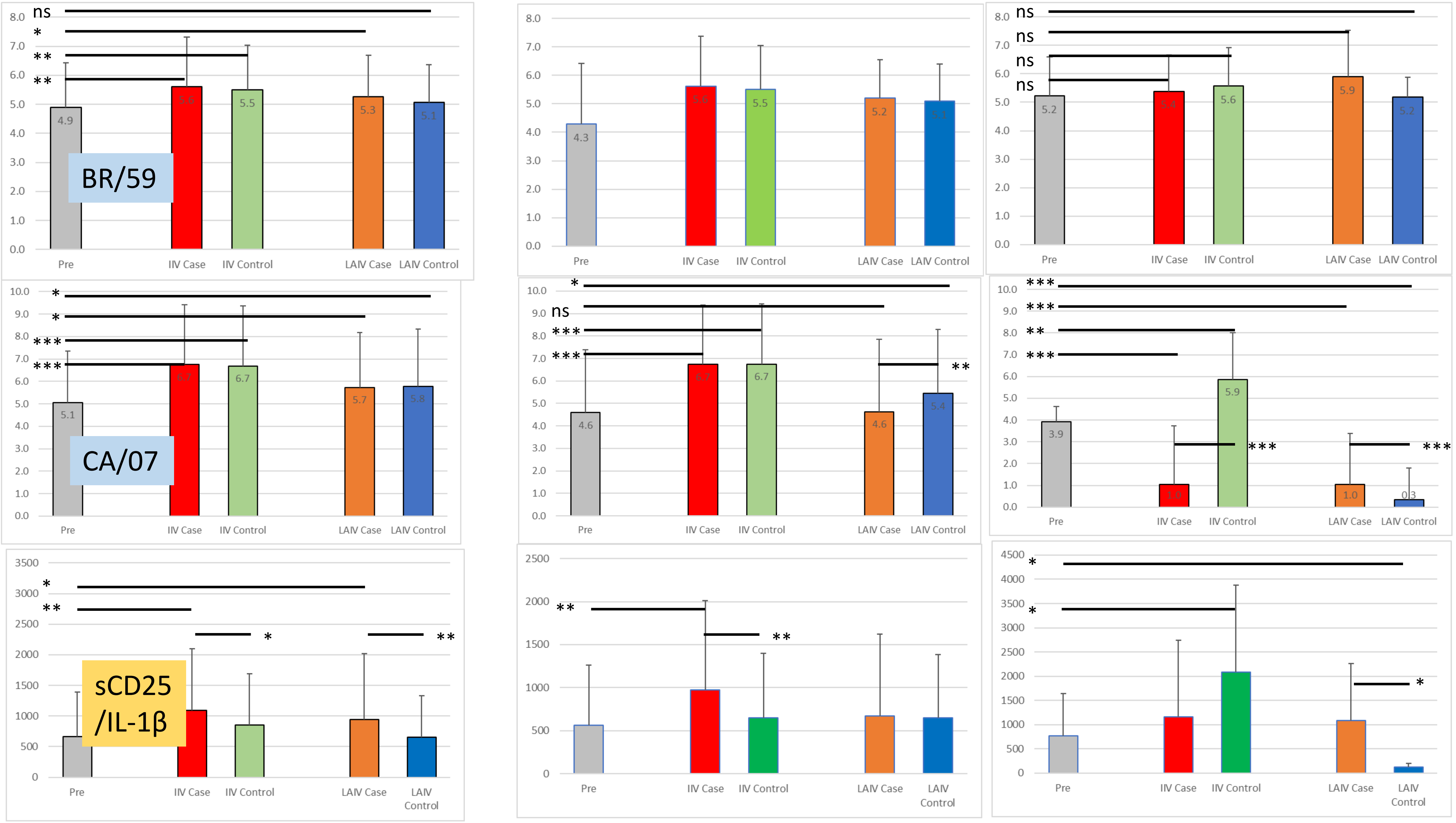

Supplementary Figure 4

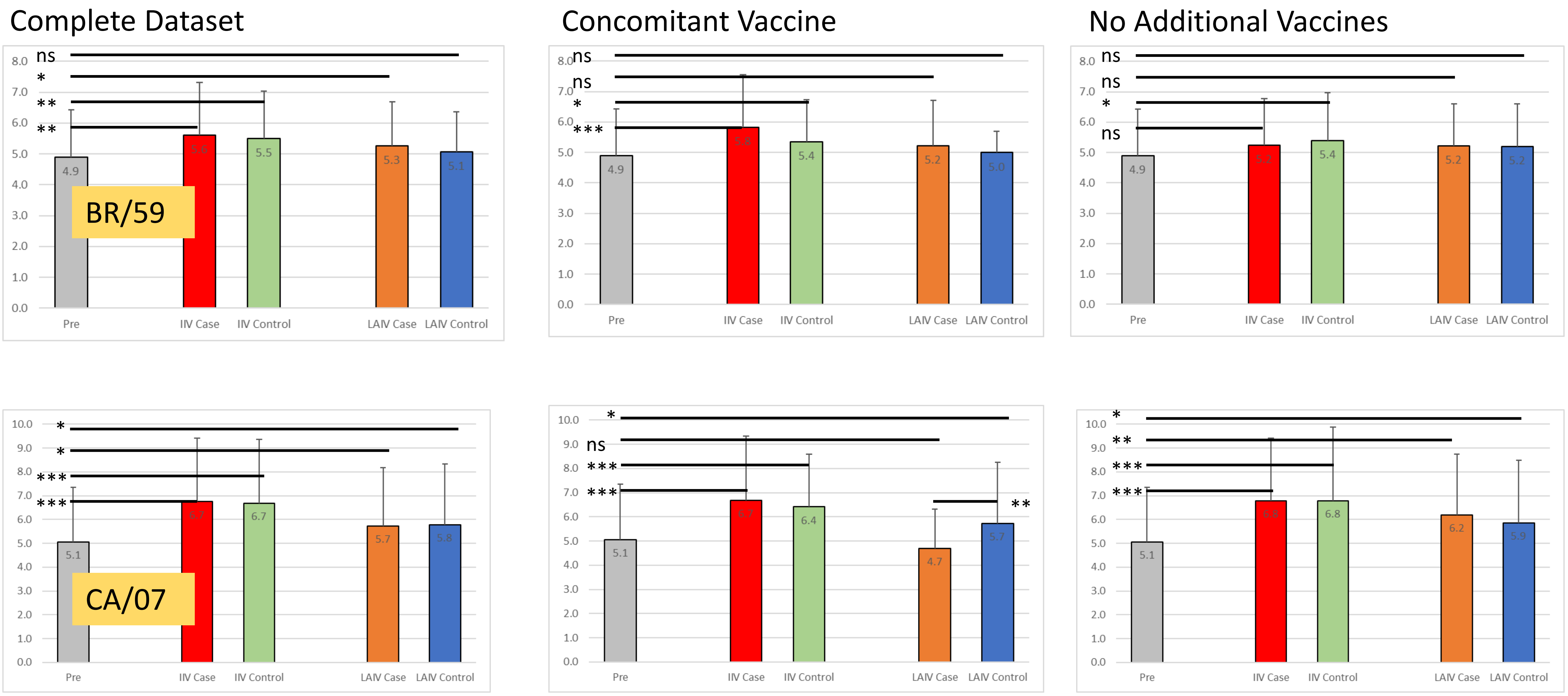

Supplementary Figure 4. Comparison of subgroups with or without additional vaccines with complete dataset. Hemagglutination Inhibition titers (log2) are shown for A/Brisbane/59/2007 (BR/59) and A/California/07/2009 (CA/07). The mean value for each group is shown with standard deviations. ns, not significant; *, p-value <0.05; **, p-value < 0.01; ***, p-value < 0.001.

Supplementary Figure 4 continued

Complete Dataset

Concomitant Vaccine

No Additional Vaccines

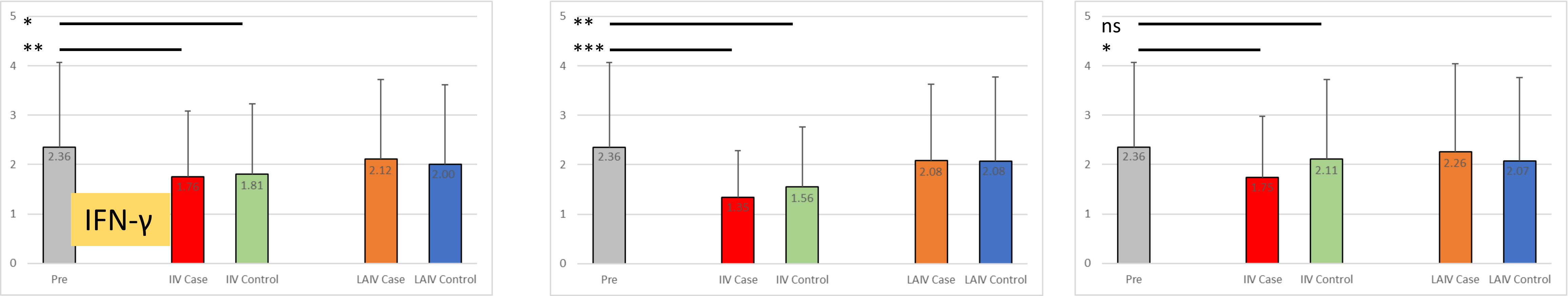

Supplementary Figure 4 continued. Comparison of subgroups with or without additional vaccines with complete dataset.
Cytokine levels (pg/mL) are shown for IFN-γ. The mean value for each group is shown with standard deviations. ns, not significant; *, p-value <0.05; **, p-value < 0.01; ***, p-value < 0.001.

Supplementary Figure 4 continued

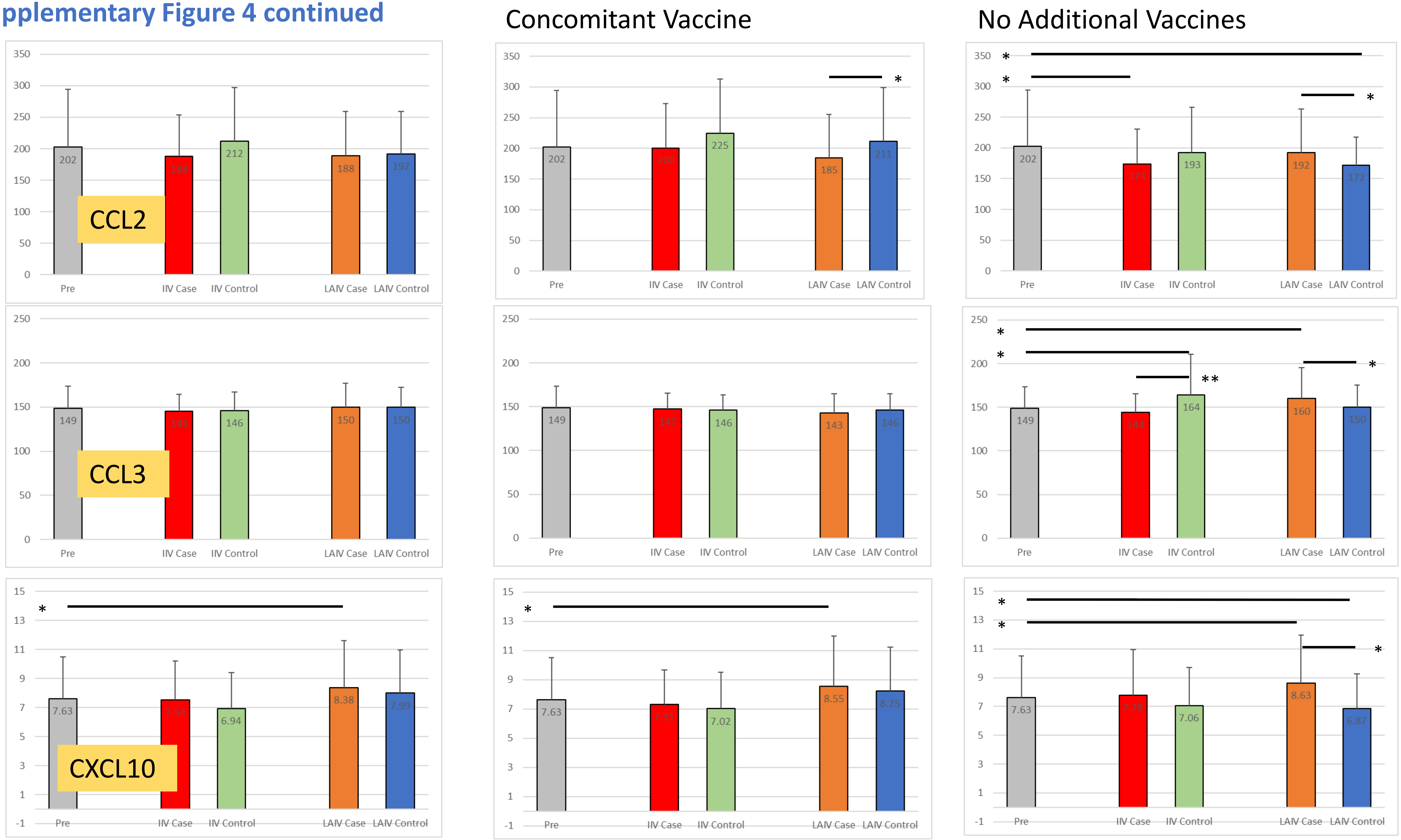

Supplementary Figure 4 continued

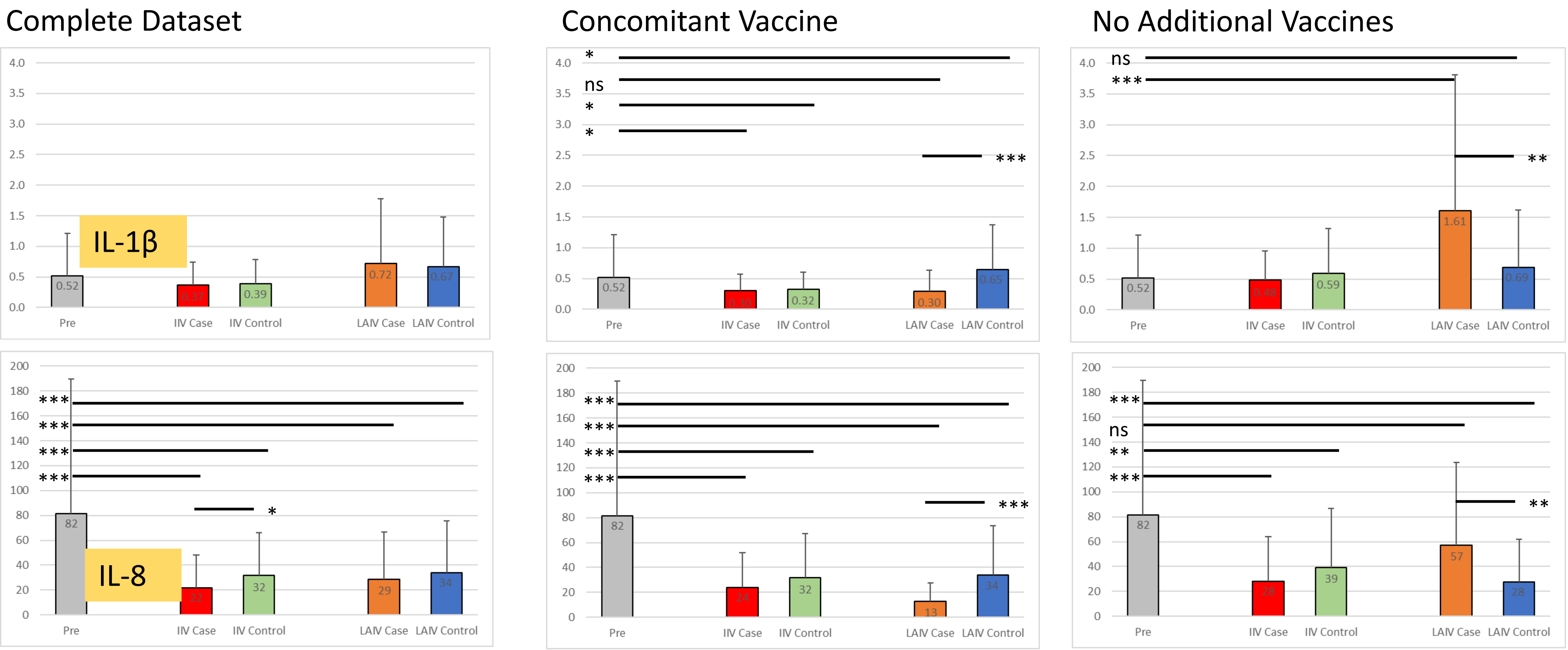

Supplementary Figure 4 continued. Comparison of subgroups with or without additional vaccines with complete dataset.
Cytokine levels (pg/mL) are shown for IL-1β and IL-8. The mean value for each group is shown with standard deviations. ns, not significant; *, p-value <0.05; **, p-value < 0.01; ***, p-value < 0.001.

Supplementary Figure 4 continued

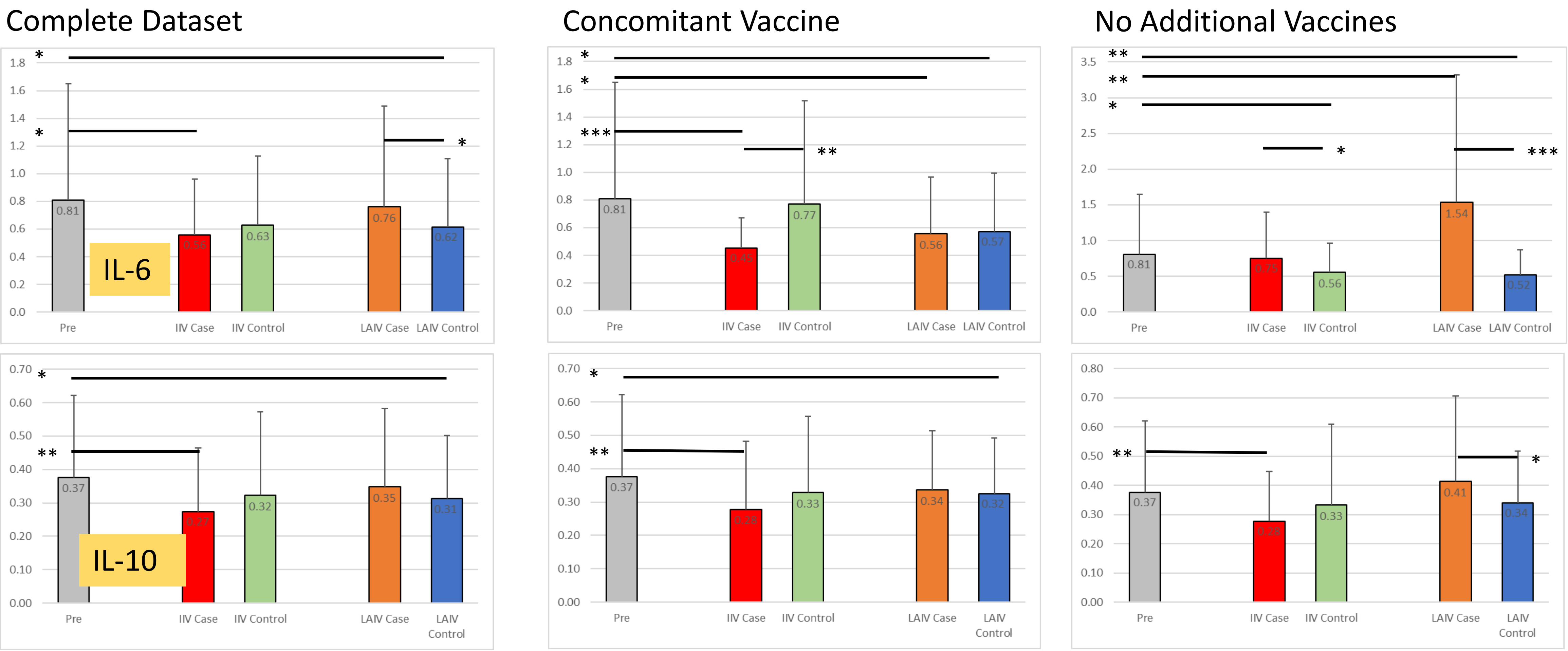

Supplementary Figure 4 continued. Comparison of subgroups with or without additional vaccines with complete dataset.
Cytokine levels (pg/mL) are shown for IL-6 and IL-10. The mean value for each group is shown with standard deviations. ns, not significant; *, p-value <0.05; **, p-value < 0.01; ***, p-value < 0.001.

Supplementary Figure 4 continued

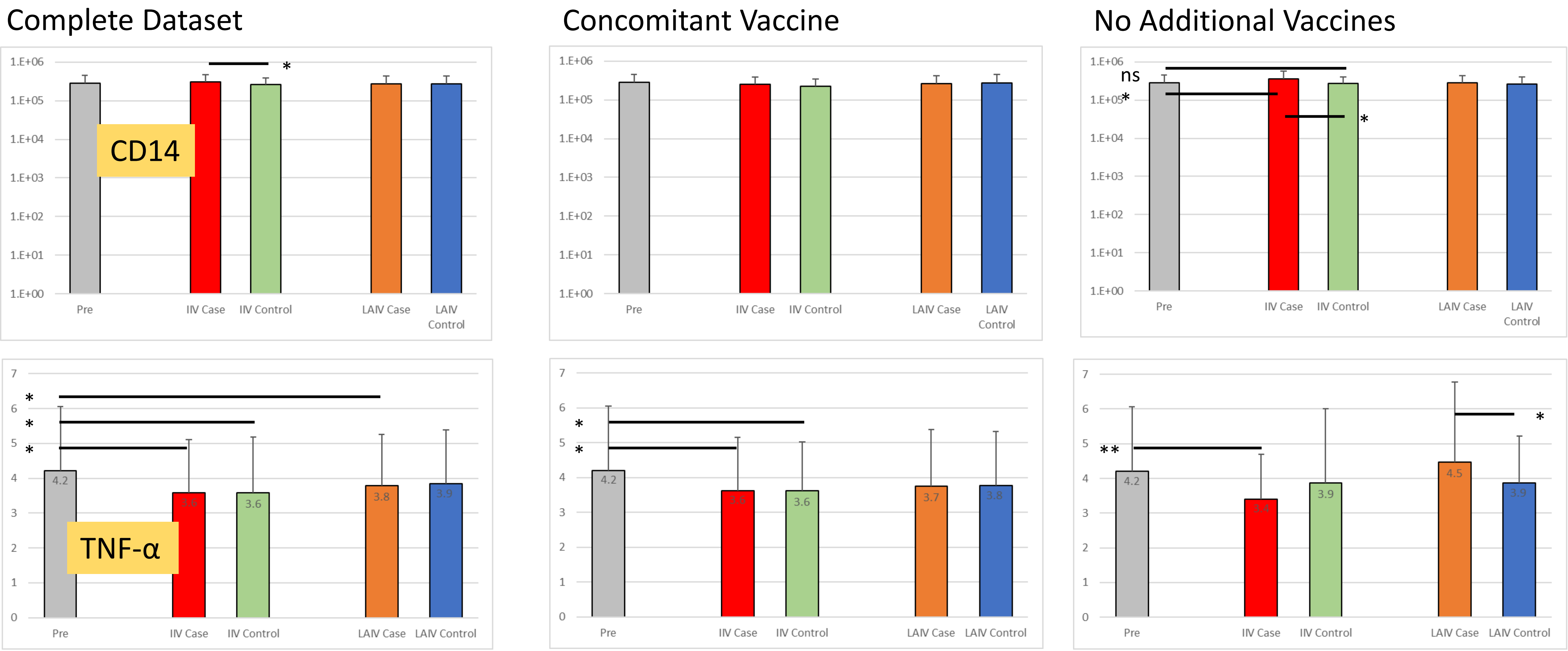

Supplementary Figure 4 continued. Comparison of subgroups with or without additional vaccines with complete dataset.
Cytokine levels (pg/mL) are shown for CD14 and TFN-α. The mean value for each group is shown with standard deviations. ns, not significant; *, p-value <0.05; **, p-value < 0.01; ***, p-value < 0.001.

Supplementary Figure 4 continued

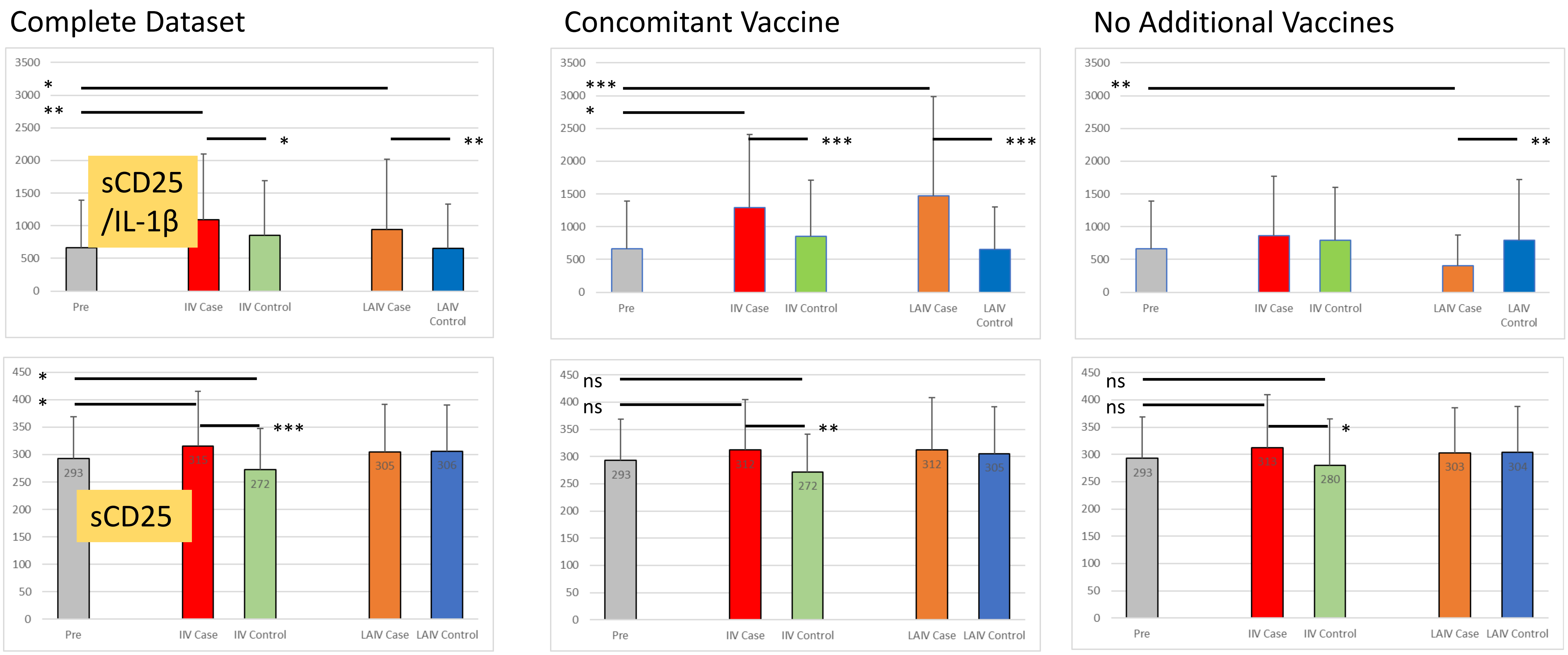

Supplementary Figure 4 continued. Comparison of subgroups with or without additional vaccines with complete dataset.
Cytokine levels (pg/mL) are shown for sCD25/IL-1β and sCD25. The mean value for each group is shown with standard deviations. ns, not significant; *, p-value <0.05; **, p-value < 0.01; ***, p-value < 0.001.
