# Supplementary material for: Post-vaccination serum cytokines levels correlate with breakthrough influenza infections
Source: Sci Rep. 2023 Jan 20;13:1174. doi: 10.1038/s41598-023-28295-8 (PMC9857916; doi:10.1038/s41598-023-28295-8)
Supplement: Supplementary file 2 — Supplementary Tables. [file 41598_2023_28295_MOESM2_ESM.docx]

| Characteristics | Pre-vaccination group  (n = 87) | IIV Case group  (n = 106) | Matched IIV Control group  (n = 106) | LAIV Case group  (n = 150) | Matched LAIV Control group  (n = 150) |
| --- | --- | --- | --- | --- | --- |
| Age  mean (SD)  min-max | 25 (7)  18-47 | 26 (7)  18-60 | 27 (9)  17-57 | 25 (6)  18-44 | 27 (7)  18-47 |
| Blood draw, days post-vaccination  Mean, (SD) | -119 (42) | 10 (7) | 10 (7) | 9 (6) | 8 (7) |
| Sex  Male, n (%)  Female, n (%) | 77 (89)  10 (11) | 90 (85)  16 (15) | 92 (87)  14 (13) | 120 (80)  30 (20) | 141 (94)  9 ( 6) |
| Race  White, n (%)  Black, n (%)  Other, n (%)  Unknown, n (%) | 54 (62)  22 (25)  8 ( 9)  3 ( 3) | 73 (69)  16 (15)  8 ( 8)  9 ( 8) | 71 (67)  19 (18)  10 ( 9)  6 ( 6) | 86 (57)  34 (23)  21 (14)  9 ( 6) | 99 (66)  31 (21)  11 ( 7)  9 ( 6) |

Supplementary Table 1. Population Characteristics.

| Analyte^1^ | Pre-vaccination^2^ group  (n = 87) | IIV Case group^2,3^  (n = 106) | Matched IIV^2,3,4^ Control group  (n = 106) | LAIV Case^2,3^ group  (n = 150) | Matched LAIV^2,3,4^ Control group  (n = 150) |
| --- | --- | --- | --- | --- | --- |
| BAFF  mean (SD)  n | 372 (94)  83 | 356 (95)*  94 | 342 (86)  95 | 368 (88)  141 | 368 (80)  140 |
| CCL2  mean (SD)  n | 202 (92)  81 | 188 (66)  93 | 212 (85) †  99 | 188 (71)  131 | 192 (68)  137 |
| CCL3  mean (SD)  n | 149 (25)  68 | 145 (19)  87 | 146 (21)  83 | 150 (27)  122 | 150 (22)  123 |
| sCD14  mean (SD)  n | 2.8E+5 (1.7E+5)  44 | 3.1E+5 (1.7E+5)  74 | 2.6E+5 (1.7E+5)  76 | 2.8E+5 (1.6E+5)  92 | 2.8E+5 (1.7E+5)  97 |
| sCD25  mean (SD)  n | 293 (77)  78 | 315 (100)**  93 | 272 (75)**, †††  90 | 305 (86)  133 | 306 (84)  136 |
| sCD163  mean (SD)  n | 2.3E+5 (1.1E+5)  80 | 2.1E+5 (0.9E+5)  93 | 2.2E+5 (0.9E+5)  97 | 2.2E+5 (0.9E+5)  134 | 2.3E+5 (0.9E+5)  132 |
| CRP  mean (SD)  n | 1.2E+5 (0.6E+5)  77 | 1.3E+5 (0.7E+5)  95 | 1.3E+5 (0.6E+5)  96 | 1.2E+5 (0.6E+5)  143 | 1.2E+5 (0.6E+5)  136 |
| CXCL10  mean (SD)  n | 7.6 (2.9)  79 | 7.5 (2.7)  90 | 6.9 (2.5)  91 | 8.4 (3.2) *  130 | 8.0 (3.0)  133 |
| IFNγ  mean (SD)  n | 2.4 (1.7)  76 | 1.8 (1.3) **  99 | 1.8 (1.4) *  93 | 2.1 (1.6)  140 | 2.0 (1.6)  145 |
| IL-1β  mean (SD)  n | 0.52 (0.69)  66 | 0.37 (0.37)  87 | 0.39 (0.38)  81 | 0.72 (1.07)  121 | 0.67 (0.81)  122 |
| IL-6  mean (SD)  n | 0.81 (0.84)  68 | 0.56 (0.41) *  84 | 0.63 (0.50)  84 | 0.76 (0.73)  120 | 0.62 (0.49) *, †  121 |
| sIL-6R  mean (SD)  n | 1.6E+4 (3E+3)  74 | 1.5E+4 (3E+3)  91 | 1.6E+4 (3E+3)  95 | 1.6E+4 (4E+3)  135 | 1.6E+4 (3E+3)  134 |
| IL-8  mean (SD)  n | 82 (108)  72 | 22 (26) ***  87 | 32 (35) ***,†  84 | 29 (38)***  121 | 34 (42) ***  123 |
| IL-10  mean (SD)  n | 0.37 (0.25)  69 | 0.27 (0.19)  90 | 0.32 (0.25)  91 | 0.35 (0.23)  134 | 0.31 (0.19)  125 |
| TNFα  mean (SD)  n | 4.2 (1.8)  74 | 3.6 (1.5)  94 | 3.6 (1.6)  90 | 3.8 (1.5)  126 | 3.9 (1.5)  127 |

Supplementary Table 2. Mean Cytokine Values (and Standard Deviation) for Groups.

^1^, for each analyte the number (n) of samples (excluding outliers) use to calculate the mean and standard deviation (SD) for each group is shown; ^2^, mean analyte concentration and SD are shown as pg/mL; ^3^, instances when all four post-vaccination groups differ from the pre-vaccination group with p-values less than 0.05 are indicated (*, p < 0.05; ***, p < 0.001) ; ^4^, differences between respective Case and Control groups with p-values less than 0.05 are indicated (†, p < 0.05; †††, p < 0.001).

| Analyte^1^ | Pre-vaccination^2^ group  (n = 87) | IIV Case group^2,3^  (n = 106) | Matched IIV^2,3,4^ Control group  (n = 106) | LAIV Case^2,3^ group  (n = 150) | Matched LAIV^2,3,4^ Control group  (n = 150) |
| --- | --- | --- | --- | --- | --- |
| BAFF  mean (SD)  n | 372 (94)  83 | 389 (113)  53 | 353 (88) †  55 | 356 (85)  71 | 357 (84)  69 |
| CCL2  mean (SD)  n | 202 (92)  81 | 174 (57) *  47 | 193 (74)  55 | 192 (72)  65 | 172 (46) **, †  67 |
| CCL3  mean (SD)  n | 149 (25)  68 | 144 (21)  44 | 164 (46) *, ††  51 | 160 (35) *  60 | 150 (25) †  61 |
| sCD14  mean (SD)  n | 2.8E+5 (1.7E+5)  44 | 3.1E+5 (1.7E+5)  74 | 2.6E+5 (1.7E+5)  76 | 2.8E+5 (1.6E+5)  92 | 2.8E+5 (1.7E+5)  97 |
| sCD25  mean (SD)  n | 293 (77)  78 | 313 (97)  49 | 280 (85) †  52 | 303 (83)  67 | 304 (84)  68 |
| sCD163  mean (SD)  n | 2.3E+5 (1.1E+5)  80 | 2.1E+5 (0.9E+5)  49 | 2.3E+5 (1.1E+5)  56 | 2.3E+5 (0.9E+5)  67 | 2.3E+5 (0.7E+5)  60 |
| CRP  mean (SD)  n | 1.2E+5 (0.6E+5)  77 | 1.1E+5 (0.7E+5)  47 | 1.2E+5 (0.7E+5)  54 | 1.1E+5 (0.6E+5)  68 | 1.3E+5 (0.7E+5)  71 |
| CXCL10  mean (SD)  n | 7.6 (2.9)  79 | 7.5 (2.7)  47 | 6.9 (2.5)  51 | 8.6 (3.3) *  66 | 6.9 (2.4)* , †††  62 |
| IFNγ  mean (SD)  n | 2.4 (1.7)  76 | 1.8 (1.3) *  49 | 2.1 (1.6)  55 | 2.3 (1.8)  68 | 2.1 (1.7)  74 |
| IL-1β  mean (SD)  n | 0.52 (0.69)  66 | 0.48 (0.47)  45 | 0.59 (0.73)  46 | 1.61 (2.2) ***  59 | 0.69 (0.93) ††  61 |
| IL-6  mean (SD)  n | 0.81 (0.84)  68 | 0.56 (0.41) *  44 | 0.63 (0.50) *, †  47 | 0.76 (0.73) **  63 | 0.62 (0.49) **, †††  59 |
| sIL-6R  mean (SD)  n | 1.6E+4 (3E+3)  74 | 1.5E+4 (3E+3)  47 | 1.7E+4 (3E+3)  53 | 1.5E+4 (4E+3)  66 | 1.5E+4 (3E+3)  66 |
| IL-8  mean (SD)  n | 82 (108)  72 | 28 (36) ***  47 | 39 (47) **  49 | 57 (66)*  63 | 28 (34) ***, ††  59 |
| IL-10  mean (SD)  n | 0.37 (0.25)  69 | 0.28 (0.17) **  45 | 0.33 (0.28)  51 | 0.41 (0.29)  68 | 0.34 (0.18) †  63 |
| TNFα  mean (SD)  n | 4.2 (1.8)  74 | 3.4 (1.3) **  46 | 3.9 (2.1)  52 | 4.5 (2.3)  62 | 3.9 (1.4) †  60 |

Supplementary Table 3. Mean Cytokine Values (and Standard Deviation) Single Vaccination Subgroup.

^1^, for each analyte the number (n) of samples (excluding outliers) use to calculate the mean and standard deviation (SD) for each group is shown; ^2^, mean analyte concentration and SD are shown as pg/mL; ^3^, instances when all four post-vaccination groups differ from the pre-vaccination group with p-values less than 0.05 are indicated (*, p < 0.05; **, p < 0.01; ***, p < 0.001) ; ^4^, differences between respective Case and Control groups with p-values less than 0.05 are indicated (†, p < 0.05; ††, p < 0.01; †††, p < 0.001).

| Analyte^1^ | Pre-vaccination^2^ group  (n = 87) | IIV Case group^2,3^  (n = 106) | Matched IIV^2,3,4^ Control group  (n = 106) | LAIV Case^2,3^ group  (n = 150) | Matched LAIV^2,3,4^ Control group  (n = 150) |
| --- | --- | --- | --- | --- | --- |
| BAFF  mean (SD)  n | 372 (94)  83 | 351 (86)  53 | 336 (82) **  55 | 398 (88) *  75 | 376 (81) †  93 |
| CCL2  mean (SD)  n | 202 (92)  81 | 200 (73)  52 | 225 (88)  60 | 185 (70)  72 | 211 (88) †  97 |
| CCL3  Mean (SD)  n | 149 (25)  68 | 147 (17)  47 | 146 (17)  50 | 143 (22)  68 | 146 (19)  81 |
| sCD14  mean (SD)  n | 2.8E+5 (1.7E+5)  44 | 2.6E+5 (1.3E+5)  40 | 2.3E+5 (1.2E+5)  38 | 3.5E+5 (1.5E+5)  50 | 2.6E+5 (1.7E+5)  64 |
| sCD25  mean (SD)  n | 293 (77)  78 | 312 (92)  48 | 272 (69) ††  54 | 312 (96)  74 | 305 (86)  89 |
| sCD163  mean (SD)  n | 2.3E+5 (1.1E+5)  80 | 2.3E+5 (1.0E+5)  51 | 2.3E+5 (0.9E+5)  59 | 2.1E+5 (0.8E+5)  71 | 2.3E+5 (1.0E+5)  89 |
| CRP  mean (SD)  n | 1.2E+5 (0.6E+5)  77 | 1.4E+5 (0.7E+5)  52 | 1.3E+5 (0.6E+5)  56 | 1.2E+5 (0.6E+5)  81 | 1.1E+5 (0.6E+5)  88 |
| CXCL10  mean (SD)  n | 7.6 (2.9)  79 | 7.3 (2.4)  49 | 7.0 (2.5)  54 | 8.6 (3.4) *  72 | 8.3 (3.0)  89 |
| IFNγ  mean (SD)  n | 2.4 (1.7)  76 | 1.4 (1.4) ***  49 | 1.6 (1.6) **  52 | 2.1 (1.5)  80 | 2.1 (1.7)  95 |
| IL-1β  mean (SD)  n | 0.52 (0.69)  66 | 0.30 (0.27) *  47 | 0.32 (0.28) *  47 | 0.30 (0.34) *  68 | 0.65 (0.73) ††  82 |
| IL-6  mean (SD)  n | 0.81 (0.84)  68 | 0.45 (0.22) ***  46 | 0.77 (0.75) ††  51 | 0.56 (0.41) *  67 | 0.57 (0.42) *  80 |
| sIL-6R  mean (SD)  n | 1.6E+4 (3E+3)  74 | 1.6E+4 (3E+3)  48 | 1.6E+4 (2E+3)  49 | 1.6E+4 (4E+3)  71 | 1.6E+4 (2E+3)  76 |
| IL-8  mean (SD)  n | 82 (108)  72 | 24 (28) ***  48 | 32 (35) ***  50 | 13 (15) ***  65 | 34 (40) ***, †††  82 |
| IL-10  mean (SD)  n | 0.37 (0.25)  69 | 0.28 (0.21) **  50 | 0.33 (0.23)  54 | 0.34 (0.18)  67 | 0.32 (0.17)  76 |
| TNFα  mean (SD)  n | 4.2 (1.8)  74 | 3.6 (1.5) *  52 | 3.6 (1.4) *  53 | 3.7 (1.6)  79 | 3.8 (1.6)  90 |

Supplementary Table 4. Mean Cytokine Values (and Standard Deviation) for Subgroup Receiving Additional Vaccines.

^1^, for each analyte the number (n) of samples (excluding outliers) use to calculate the mean and standard deviation (SD) for each group is shown; ^2^, mean analyte concentration and SD are shown as pg/mL; ^3^, instances when all four post-vaccination groups differ from the pre-vaccination group with p-values less than 0.05 are indicated (*, p < 0.05; **, p < 0.01; ***, p < 0.001) ; ^4^, differences between respective Case and Control groups with p-values less than 0.05 are indicated (†, p < 0.05; ††, p < 0.01; †††, p < 0.001).
